# Cold case: The disappearance of Egypt bee virus, a fourth distinct master strain of deformed wing virus linked to honeybee mortality in 1970’s Egypt

**DOI:** 10.1186/s12985-022-01740-2

**Published:** 2022-01-15

**Authors:** Joachim R. de Miranda, Laura E. Brettell, Nor Chejanovsky, Anna K. Childers, Anne Dalmon, Ward Deboutte, Dirk C. de Graaf, Vincent Doublet, Haftom Gebremedhn, Elke Genersch, Sebastian Gisder, Fredrik Granberg, Nizar J. Haddad, Rene Kaden, Robyn Manley, Jelle Matthijnssens, Ivan Meeus, Hussein Migdadi, Meghan O. Milbrath, Fanny Mondet, Emily J. Remnant, John M. K. Roberts, Eugene V. Ryabov, Noa Sela, Guy Smagghe, Hema Somanathan, Lena Wilfert, Owen N. Wright, Stephen J. Martin, Brenda V. Ball

**Affiliations:** 1 Department of Ecology, Swedish University of Agricultural Sciences, 750-07 Uppsala, Sweden; 2 Hawkesbury Institute for the Environment, Western Sydney University, Locked Bag 1797, Renrith, NSW 2751 Australia; 3 School of Environment and Life Sciences, University of Salford, Manchester, M5 4WT UK; 4 Institute of Plant Protection, The Volcani Center, PO Box 15159, 7528809 Rishon Lezion, Israel; 5 Bee Research Laboratory, Beltsville Agricultural Research Center, USDA, Beltsville, MD 20705 USA; 6 Abeilles et Environnement, INRAE, 84914 Avignon, France; 7 Department of Microbiology, Immunology and Transplantation, Rega Institute, Laboratory for Clinical and Epidemiological Virology, University of Leuven, 3000 Leuven, Belgium; 8 Max Planck Institute of Immunobiology and Epigenetics, Stübeweg 51, 79108 Freiburg, Germany; 9 Laboratory of Molecular Entomology and Bee Pathology, Ghent University, 9000 Ghent, Belgium; 10 College of Life and Environmental Sciences, University of Exeter, Penryn, TR10 9FE UK; 11 Institute of Evolutionary Ecology and Conservation Genomics, University of Ulm, Albert-Einstein-Allee 11, 89081 Ulm, Germany; 12 Tigray Agricultural Research Institute, P.O. Box 492, Mekelle, Ethiopia; 13 Institut Für Mikrobiologie Und Tierseuchen, Fachbereich Veterinärmedizin, Freie Universität Berlin, Berlin, Germany; 14 Department of Molecular Microbiology and Bee Diseases, Institute for Bee Research, Hohen Neuendorf, Germany; 15 Department of Biomedical Sciences and Veterinary Public Health, Swedish University of Agricultural Sciences, 750-07 Uppsala, Sweden; 16 Bee Research Department, National Agricultural Research Center, Baq’a, Jordan; 17 Present Address: Clinical Microbiology, Department of Medical Sciences, Uppsala University, 753 09 Uppsala, Sweden; 18 Laboratory of Agrozoology, Department of Plants and Crops, Ghent University, Coupure Links 653, 9000 Ghent, Belgium; 19 Behaviour, Ecology and Evolution (BEE) Lab, School of Life and Environmental Sciences, The University of Sydney, Camperdown, 2006 Australia; 20 Commonwealth Scientific and Industrial Research Organisation, Canberra, 2601 Australia; 21 School of Biology, Indian Institute of Science Education and Research, Thiruvananthapuram, Kerala 695551 India; 22 Centre for Research in Animal Behaviour, College of Life and Environmental Sciences, University of Exeter, Exeter, EX4 4QG UK; 23 Rothamsted Research, Harpenden, Hertfordshire, AL5 2JQ UK; 24 Present Address: Department of Vector Biology, Liverpool School of Tropical Medicine, Liverpool, L3 5QA UK

**Keywords:** Egypt bee virus, Deformed wing virus, Master strain, *Varroa destructor*, Honeybee, *Apis mellifera*, Western blot, RNA sequencing, Bioinformatic screening

## Abstract

In 1977, a sample of diseased adult honeybees (*Apis mellifera*) from Egypt was found to contain large amounts of a previously unknown virus, Egypt bee virus, which was subsequently shown to be serologically related to deformed wing virus (DWV). By sequencing the original isolate, we demonstrate that Egypt bee virus is in fact a fourth unique, major variant of DWV (DWV-D): more closely related to DWV-C than to either DWV-A or DWV-B. DWV-A and DWV-B are the most common DWV variants worldwide due to their close relationship and transmission by *Varroa destructor*. However, we could not find any trace of DWV-D in several hundred RNA sequencing libraries from a worldwide selection of honeybee, varroa and bumblebee samples. This means that DWV-D has either become extinct, been replaced by other DWV variants better adapted to varroa-mediated transmission, or persists only in a narrow geographic or host range, isolated from common bee and beekeeping trade routes.

**Supplementary Information:**

The online version contains supplementary material available at 10.1186/s12985-022-01740-2.

## Introduction

For most of the twentieth century, the bee unit at the Rothamsted Research in England was the de facto worldwide reference point for honeybee pathology, due to its research and diagnostic expertise. As a result, the unit regularly received bee samples from around the world, from colonies with unusual symptoms or unclear causes of mortality. These samples fed the research programme, international network and diagnostic capabilities of the unit. One such sample, sent from Egypt during spring 1977, led to the discovery and initial characterization of a new bee virus, named Egypt bee virus (EBV) after its geographic origin [3]. In those days, honeybee virology was a rather niche topic with little practical relevance, except for the occasional case of paralysis or sacbrood, which tended to solve itself with a change of season or scenery. This changed dramatically with the initial adaptation, speciation [2] and subsequent worldwide dissemination of the ectoparasitic mite *Varroa destructor* from its original host (*Apis cerana*) to its current host (*A. mellifera*) during the second half of the twentieth century [68], possibly as an unintended consequence of the large-scale introduction of commercial beekeeping with *A. mellifera* to Asia after WW-II [11, 70], during the post-colonial agricultural transformation, or Green Revolution [52] of Asia. This mite is highly efficient at transmitting and facilitating several bee viruses [73], resulting in the rapid decline of a bee colony unless active measures are taken to keep the mite population under control (for a recent review, see [68].

Of the many viruses identified in honeybees (Beaurepaire et al. 2020), the main varroa-transmitted virus worldwide is Deformed wing virus (DWV; [14], the last major virus to be identified by the Rothamsted bee unit [41]. Three major genetic variants of DWV have thus far been identified: DWV-A [18, 33], DWV-B [48], and DWV-C [46]. DWV-A and DWV-B have slightly different effects on replication, transmission, virulence, pathology, host range and individual and collective bee mortality and functionality [21, 22, 41, 62] that are determinative for their continued coexistence in relation to different bee and mite management practices [68]. Natural and artificial recombinants between these strains [12, 45] have made it possible to link some of these characteristics to particular regions of the DWV genome [21, 60, 62].

Here, we demonstrate that EBV is in fact a fourth unique, major variant of DWV: more closely related to DWV-C than to either DWV-A or DWV–B. We therefore propose to re-assign EBV as DWV major variant D (DWV-D), in continuation of the current DWV strain classification system. However, we could not find any trace of this variant in several hundred RNA sequencing libraries from honeybees, varroa mites, and other pollinators worldwide. This means that this variant has either become extinct, been overtaken and replaced by other DWV variants better adapted to new transmission routes, or persists only in a narrow geographic or host range isolated from common bee and beekeeping trading routes.

## Materials and methods

### Sample origin

The provenance of the Egypt bee virus sample is described in Bailey et al. [3]. The original field sample consisted of diseased adult bees sent during spring 1977 from Egypt to Rothamsted Research in England for pathological analysis. A purified extract from 30 bees that contained large numbers of 30 nm isometric picornavirus-like particles, a third of which appeared to be empty, failed to react to antisera raised against the known bee viruses at the time (ABPV, BQCV, CBPV, KBV, SBPV, and SBV) in immunodiffusion tests [3]. During June 1977, the extract was propagated at 10^–4^ dilution in white-eyed pupae (see [15] in the presence of antiserum against CBPV, SBV, and BQCV, which is an effective method to prevent co-amplification of contaminating viruses [15]. Purified extracts from several of these pupae again failed to react against any of the antisera against the known viruses, despite containing large amounts of similar particles as in the primary extract, including the high proportion of empty particles. The remaining pupae were freeze-dried and stored at room temperature during the following decades. Drying is a well-established method for the long-term preservation of virus samples, allowing the recovery of live virus even from centuries-old herbarium specimens [17, 26].

### Serology

During 1977, a polyvalent antiserum was developed in rabbits against the propagated EBV virus preparation by intramuscular injection of 1 mg purified virus, followed by two weekly booster injections of 0.1 mg virus emulsified with an equal volume of Freund’s complete adjuvant. Blood serum was collected at weekly intervals and titrated against the virus preparation until the highest titer was reached (e.g. [1, 4]. A similar antiserum was developed in 1985 against deformed wing virus (DWV), after propagating an extract from deformed adult bees collected in 1982 in Japan.

### SDS-PAGE and Western blot

The original propagated virus preparations were denatured and fractionated on a discontinuous SDS-PAGE gel alongside a series of molecular weight markers of known size and visualized with Coomassie Blue staining [1, 3, 29]. The molecular mass of the viral proteins was estimated from a calibration curve established by the migration distances of the molecular weight markers [8]. The proteins were transferred to nitrocellulose membrane (Western blot) according to Allen and Ball [1] (see also [29]). The Western blots were incubated for 16 h at room temperature with a 1/1000 dilution of primary antibody in phosphate-buffered saline phosphate (PBS) containing 1% Tween-20 and 5% fat-free powdered milk (PBS-TM). The membrane was washed 6 × 7 min with PBS-TM and incubated 2 h at room temperature with a 1/4000 dilution of an anti-rabbit-IgG antibody covalently linked to alkaline phosphatase (ThermoFisher: Waltham, MA, USA) in PBSTM. The membranes were washed 5 × 7 min with PBS and developed with 5% nitroblue tetrazolium and 5% 5-Bromo-4-Chloro-3-Indolyl Phosphate in 0.1M TRIS(9.5)/0.1M NaCl/50 mM MgCl_2_ [1].

### RNA extraction & cDNA synthesis

During June 2009, and again separately during February 2017, RNA was extracted from one of the freeze-dried pupae using the RNA cleanup protocol of the RNeasy kit (Qiagen: Hilden, Germany), after first manually homogenizing the pupa in 100 μL sterile water, finally eluting the total RNA in 50 μL sterile water. The RNA was converted to cDNA using the SuperScript-III™ kit (ThermoFisher: Waltham, MA, USA), following the manufacturer’s protocols. Each cDNA synthesis reaction contained 4 μL RNA (~ 1 μg) and 0.2 μM cDNA primer in a 20 μL volume. The RNA was denatured for 5 min at 65 °C in the presence of the cDNA primer, after which buffer and SuperScript-III were added. The reaction was incubated for 60 min at 50 °C, following which the reaction was terminated by diluting tenfold in TE (pH 7.0) and incubated for 5 min at 95 °C. In 2009, the cDNA was synthesized using eight different cDNA primers covering the entire DWV genome and designed to amplify both DWV-A and DWV-B genomes (Additional file 1: Table S1). In 2017 the cDNA was synthesized using random hexamers.

### PCR amplification

The cDNA was amplified using the iTaq system (Bio-Rad Laboratories: Hercules, CA, USA) in 50 μL volumes containing 10 μL diluted cDNA and 0.3 μM each of forward and reverse primers specific for eight overlapping fragments (A, B, C, D, E, F, G, and H) of between ~ 700 and ~ 1700 bp each, covering the entire DWV genome (Additional file 1: Table S1; Additional file 1: Figure S1) with the following thermo-cycling profile: 95 °C:1 min – 35 × [95 °C:10 s-58°C:30 s-72°C:120 s] – 72 °C:5 min – 4 °C. In 2010, the amplification was only successful for amplicons A, D, E, and H. These fragments were sequenced using both Sanger sequencing of each separate fragment (2010) and IonTorrent sequencing of pooled fragments (2014). After the publication of the DWV-C genome [46], a new set of primers was designed, this time consensual for all three major DWV strains (Additional file 1: Table S1). With these primers, additional amplicons were produced for fragments E and G, as described above. Further EBV-specific primers were designed based on partial sequence information (see below), which yielded additional products for fragments F and G. The final fragments, B and C, were obtained after first producing randomly-primed cDNA with a First Strand cDNA Synthesis Kit (ThermoFisher: Waltham, MA, USA) from 1 µg total RNA following the manufacturer’s recommended protocol, diluting fivefold with TE(8.0) buffer and then amplifying the fragments with the EBV-specific primers (Additional file 1: Table S1) using the Phusion High-Fidelity PCR kit (ThermoFisher: Waltham, MA, USA), using the thermo-cycling profile: 98 °C:30 s-35 × [98 °C:10 s-58°C:10 s-72°C:60 s] – 72 °C:10 min – 4 °C. The PCR product size and purity were confirmed by agarose gel electrophoresis before the products were submitting for sequencing.

### Sequencing and genome assembly

The first set of fragments (A, D, E, H) were sequenced in 2010 with Sanger sequencing, producing partial sequence data with intermittent genome coverage. In 2014 these fragments were pooled and submitted to IonTorrent sequencing, in a barcoded batch with 13 similar samples. The pooled DNA fragments were fragmented using the S2 system (Covaris: Woburn, MA, USA). The fragment ends were repaired and ligated to unique barcoded adaptors using the Applied Biosystems Library builder (ThermoFisher: Waltham, MA, USA). The adaptor-linked fragments were amplified using the Ion Xpress™ Plus gDNA Fragment Library Preparation protocol and selected for a 470 bp target size range with Blue Pippin™ (Sage Science: Beverley, MA, USA). Library size and concentration were assessed by a Bioanalyzer High Sensitivity Chip (Agilent Technologies: Santa Clara, CA, USA) and the Fragment Analyzer system (Advanced Analytical Technologies: Ankeny, IA, USA). The sequencing template was prepared using the Ion PGM™ Template OT2 400 Kit on the Ion OneTouch™ 2 system (ThermoFisher: Waltham, MA, USA). Samples were then sequenced on the Ion PGM™ System with Ion PGM™ Sequencing 400 Kit on a 100 Mb Ion 316v2 chip (ThermoFisher: Waltham, MA, USA) aimed at a 400 bp read length. For the EBV sample, this resulted in 214,861 sequence reads with an average length of 310 bp. The reads were mapped onto the DWV-A (AY292384) and DWV-B (AY251269) genomes using Tmap included in TorrentSuite (v.3.6.2) with recommended parameters (ThermoFisher: Waltham, MA, USA). The consensus sequences were created using mpileup from SAMtools (v.0.1.8) [35] based on a 1000 ~ 35,000× per-base coverage for the A, D, E and H fragments. The DWV-A and DWV-B mapped consensus sequences were 99% identical at nucleotide level. In 2015, the EBV RNA sample was submitted for direct RNA sequencing, in a barcoded batch of nine RNA samples, using IonTorrent technology. The RNA samples were depleted for ribosomal RNA using the RiboZero rRNA depletion kit (Illumina, San Diego, CA, USA). The quality of the depleted RNA was checked using the Bioanalyzer RNA Pico chip (Agilent Technologies: Santa Clara, CA, USA), after which the RNA was fragmented with Ribonuclease III and ligated to adaptor sequences. The RNA sequencing libraries were constructed using the Ion Total RNA-Seq v2 kit, and were sequenced on the Ion PGM™ System with an Ion PGM™ IC 200 Kit on a 80 Mb Ion 314 chip (ThermoFisher: Waltham, MA, USA) aimed at a 200 bp read length. This resulted in 38,761 reads with an average length of 173 bp. The reads were again mapped to both the DWV-A and DWV-B reference sequences, as described above. Although the overall genomic coverage of the RNA sequencing was patchy, with moderate per-base nucleotide coverage (10 ~ 18,000× for the DWV-A mapping; 10 ~ 7000× for the DWV-B mapping), there was again high (99%) nucleotide identity between the DWV-A and DWV-B mapped sequences, and with the accumulated sequence data. It also produced new partial sequence information for several of the intervening regions. EBV fragments E, F and G produced in 2017 were sequenced using PacBio technology (Pacific BioSciences: Menlo Park, CA, USA), as part of a batch of eight barcoded samples. The PCR products were repaired using the SMRTbell™ Damage repair SPv3 kit and ligated to unique barcodes using the SMRTbell™ barcoded adapter prep kit. Sequencing template was prepared using the SMRTbell™ template prep 1.0 SPv3 kit and cleaned up using the SMRTbell™ Cleanup bead kit (Pacific BioSciences: Menlo Park, CA, USA). Sequencing fragments of around 1500 bp in size, the expected sizes of fragments E, F and G (Additional file 1: Table S1), were selected with Blue Pippin™ (Sage Science: Beverley, MA, USA). Library size and quality were checked with an Agilent Bioanalyzer (Agilent Technologies: Santa Clara, CA, USA). Sequencing was performed on a Sequel™ SMRT-Cell 1 M v2 using the Sequel™ Sequencing Kit 2.0 (Pacific BioSciences: Menlo Park, CA, USA). The data consisted of ~ 6500 sequence reads of 1500–1600 bp each, 99% of which could be mapped to the DWV-A reference genome. The reads were mapped to the DWV-A reference genome using GraphMap v.0.5.2, sorted and indexed using Samtools v.1.6 and regions with < 5× coverage were filtered out using BEDTools v.2.27.1. The nucleotide variants at each position in the remaining regions were called and counted using an in-house script. The consensus was constructed using FreeBayes v.1.1.0 for variant calling and Vcflib to determine the consensus sequence from the variants, according to the mapped positions to the DWV-A reference sequence. The per-base coverage for the E, F and G fragments was between 1000 ~ 4000×. The final fragments, B and C, were obtained and sequenced in 2020 using Sanger sequencing (Macrogen-Europe BV, Amsterdam, The Netherlands). The EBV consensus sequence was condensed from these various sequencing efforts, with the few conflicts resolved conservatively. The consensus sequence has been deposited at GenBank under accession number MT504363.

### Phylogenetic analyses

The DWV-A (AY292384), DWV-B (AY251269), DWV-C (CEND01000001) and EBV (DWV-D) consensus genome sequences were aligned to each other and to Darwin bee virus-3 (MG995697); the closest full-length outgroup sequence [57], using the CLUSTAL-Omega multiple alignment programme [63] with default parameters, on the European Bioinformatics Institute website, www.ebi.ac.uk [38]. The final alignment was checked for consistency and accuracy prior to inclusion in phylogenetic analyses. Similarity plot analysis was performed on Simplot version 3.5.1, using Hamming distance in a 200 bp sliding window and 20 step size [36]. The evolutionary history for each virus was inferred using the Maximum Likelihood method based on the Tamura-Nei model [66] as implemented by MEGA-X [31], with the trees with the highest log likelihood retained. The initial trees for the heuristic searches were obtained automatically by applying Neighbor-Joining and BioNJ algorithms to a matrix of pairwise distances estimated using the Maximum Composite Likelihood (MCL) approach, and then selecting the topology with superior log likelihood value. All positions containing gaps and missing data were excluded from the analyses. The percentage of trees in which the associated taxa clustered together was determined by bootstrap analyses involving 500 replicates. Separate phylogenetic analyses were conducted for different sections of the DWV genome, bounded by the putative proteolytic sites of the DWV polyprotein [14]. The numerical details of the phylogenetic analyses are summarized in Additional file 1: Table S2.

### Screening RNA sequencing libraries

Individual reads of a large number of RNA sequencing libraries (Additional file 1: Table S3) were differentially screened for the presence of DWV-D, by first using Bowtie2 [32] with default search parameters and high sensitivity to extract all reads with a Phred quality score rating > 33 that match DWV, and then competitively matching these reads against all four DWV master strains (DWV-A, DWV-B, DWV-C, and DWV-D) using the ‘map-to-reference’ tool in Genious [30] at medium sensitivity/fast setting for recovering multiple best-to-none matches and again a minimum read quality of Phred > 30, to ensure that only those reads that best matched the DWV-D sequence were retained. To rule out misclassification due to generic SNPs that can arise in any (DWV) quasi-species [21, 60, 72] or random sequencing errors [24] at DWV-D SNPs, a read required least three linked SNPs specific to DWV-D for a positive identification. Since many of the RNA sequencing libraries had rather small (50 nt) reads, this meant that the burden of identifying the presence of DWV-D in a sample lay primarily with the Leader protein (Lp) gene, which is the region with the highest density of SNPs and thus most likely to identify several linked SNPs specific to DWV-D on an individual 50–300 nt RNA sequencing read (Additional file 1: Table 3).

## Results

In 1982, about five years after the characterization of EBV, a sample of dead adult bees with deformed wings was sent to Rothamsted from Japan for pathological characterization. The virus was purified, and a weak cross-reaction was observed in immunodiffusion tests against the EBV antiserum (Table 1). The serological relationship between EBV and the new virus from Japan was confirmed when a new antiserum raised in 1985 against the Japanese virus also cross-reacted with EBV in immunodiffusion (Table 1). Furthermore, both antisera produced characteristic ‘spurs’ in ‘Ouchterlony’ double-immunodiffusion tests, when testing each antiserum against several antigens/viruses [50]. This spur was produced when antibodies uniquely specific to its homologous virus migrated through the precipitation line between the antibody and the heterologous virus to form a second precipitation line: the spur. Such spurs are evidence of significant divergence at protein level (antigenic epitopes) between major strains of the same virus species, as shown by the primary cross-reaction [50]. The Japanese virus was re-named deformed wing virus (DWV) because of the symptoms in the adult bees [41]. The original report on EBV did not mention any wing deformities [3], which it almost certainly would have done had wing deformities been present. Western blots of the virus preparations again confirmed the cross-reaction of both antisera, slightly more pronounced for the DWV antiserum than for the EBV antiserum, and located the bulk of the antigenic activity (and cross-reaction) to VP3, the largest capsid protein [54], with a minor contribution from VP1. Five DWV VP1 subunits form the pore through which the viral RNA is injected into the cell during infection. The DWV VP3 has a protruding P domain that attaches to receptors on the cell wall and pivots on a hinge to bring the pore into contact with the cell wall, for subsequent introduction of the viral RNA into the cell [49, 54, 64]. Therefore, both these proteins have significant protrusions on the virus particle to provide epitopes for antigenic activity. The VP2, on the other hand, is more depressed, forming the inner shell of the virus particle [54], and consequently does not present many epitopes for antigenic activity.

**Table 1:**
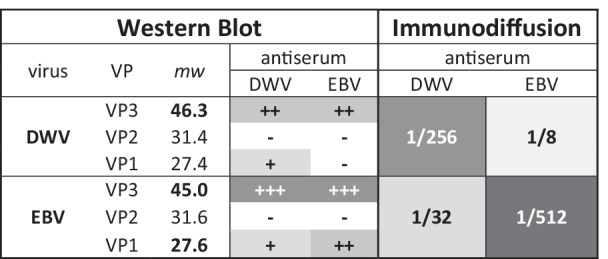
Serological analyses (1 column)

Results of serological tests comparing DWV and EBV virus preparations against their respective antisera. Left side panel concerns the cumulative results of several Western Blotting experiments. VP1, VP2, and VP3 refer to the three major structural proteins of DWV/EBV, with their molecular weights (*mw*) given in kDa. The relative intensity of Coomassie staining on SDS-PAGE of the three major proteins is indicated by bold type. The intensity of the reaction of each VP to the antisera is given on a progressive scale, from – to +++ , indicated by the intensity of the color. The right side panel concerns the results of the immunodiffusion test. The numbers indicate the highest dilution of antisera that still gives a visible precipitation line between the antigen (virus preparation) and the antisera. As for the Western Blot, a stronger reaction is indicated by a deeper color

**Fig. 1:**
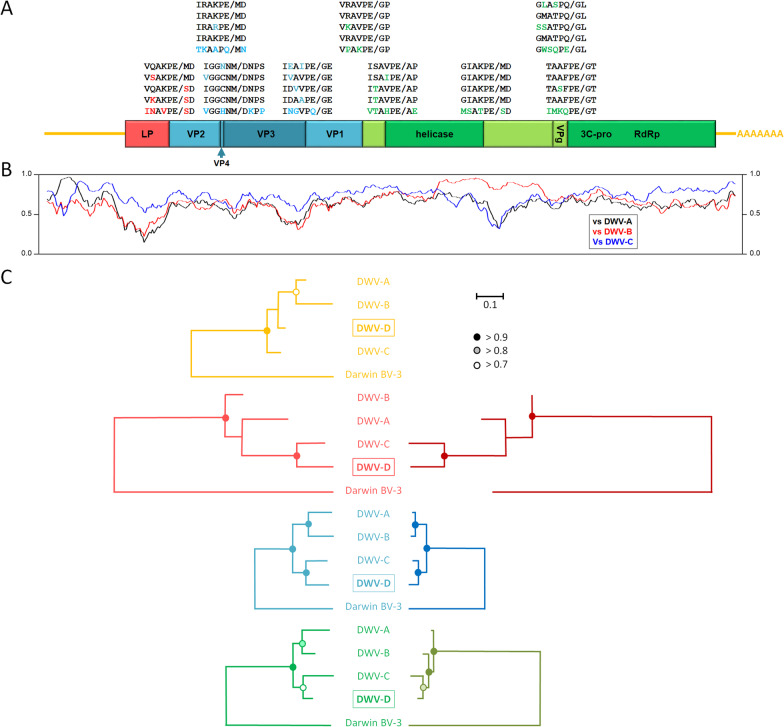
Genome organization and phylogenetic analyses (2 columns). **A** Map of the DWV-D genome showing the location and size of the major genomic regions: 5’ UTR and 3’ UTR (yellow), Lp gene (red), capsid proteins VP1, VP2, VP3 and VP4 (blue), and the non-structural proteins (green), including the helicase, VPg, 3C-protease and RdRp genes. Shown above the genome map are the conserved protease cleavage sites for DWV-A, DWV-B, DWV-C, DWV-D, and Darwin bee virus-3 for processing the polyprotein into functional units. **B** Plot of the nucleotide similarity between DWV-D and either DWV-A (black), DWV-B (red) or DWV-C (blue) across a sliding 200 bp window. **C** Phylogenetic relationships between the four major DWV variants relative to the closest known outgroup (Darwin bee virus 3) for the four major genomic regions: UTR (yellow), Lp gene (red), structural proteins (blue), and non-structural proteins (green) based on either the nucleotide sequence (left) or the amino acid sequence (right). The number of characters included in each phylogram is shown in Additional file 1: Table S2. The phylogenetic trees with the highest log likelihood (see Additional file 1: Table S2) are shown. All trees are drawn to scale, with branch lengths measured in the number of substitutions (nucleotide or amino acid) per site. The degree of confidence in the branching nodes, based on bootstrapping the alignment 500 times, is shown by the solid, shaded, and white circles. Nodes with less than 70% bootstrap support (no circle) are unreliable

The full sequence of Egypt bee virus genome was determined using a variety of sequencing approaches. The genome organization is typical for the Iflaviridae and deformed wing virus, with a rather long 5’ untranslated region followed by a single 2896 amino acid Open Reading Frame (ORF) coding for a leader protein (Lp), followed by the four capsid proteins that comprise the Iflavirus particle [54], followed by several non-structural proteins, followed by a shorter 3’ untranslated region and terminated by a natural poly-adenylated tail (Fig. 1A; [69]). The autocatalytic and 3C-protease recognition sites, where the polyprotein is cleaved into functional units [14, 69], are highly conserved (and frequently identical) between EBV and the three major DWV strains, and quite distinct from those of their closest relative, Darwin bee virus-3 (Fig. 1A). Egypt bee virus is most similar to DWV-C across most of its genome, except for a short section early in the 5’ NTR, where it is most similar to DWV-A, and an extensive section between the helicase and 3C-protease genes, where it is much more similar to DWV-B, and very distinct from DWV-A and DWV-C (Fig. 1B). These broad observations underpin the phylogenetic analyses (Fig. 1C). These show that Egypt bee virus is clearly a distinct fourth DWV master variant, DWV-D: more closely related to DWV-C than to either DWV-A or DWV-B (Fig. 1B). The topology of the phylogeny is very consistent across the major genomic regions, and between the phylogenies based on the nucleotide or amino acid sequences. The most divergent genomic region by far is the Lp gene, particularly the amino acid sequence [14, 33], while the structural protein region was the least divergent. Except for the untranslated regions, DWV-D consistently clusters with DWV-C with high confidence. Except for the Lp gene, DWV-A and DWV-B also consistently cluster together with high confidence. The most stable overall topology is two separate clusters, one containing DWV-D and DWV-C, and the other cluster containing DWV-B and DWV-A. The branching nodes not conforming to this pattern are unstable with no significant support. The high similarity of the proteolytic processing sites (Fig. 1A) implies that the 3C-proteases of the four DWV variants can almost certainly process each others’ polyproteins when part of a common quasi-species [16, 72]. Such functional complementation would allow DWV-D genomes to persist at low levels in DWV quasi-species dominated by the other strains.

**Fig. 2:**
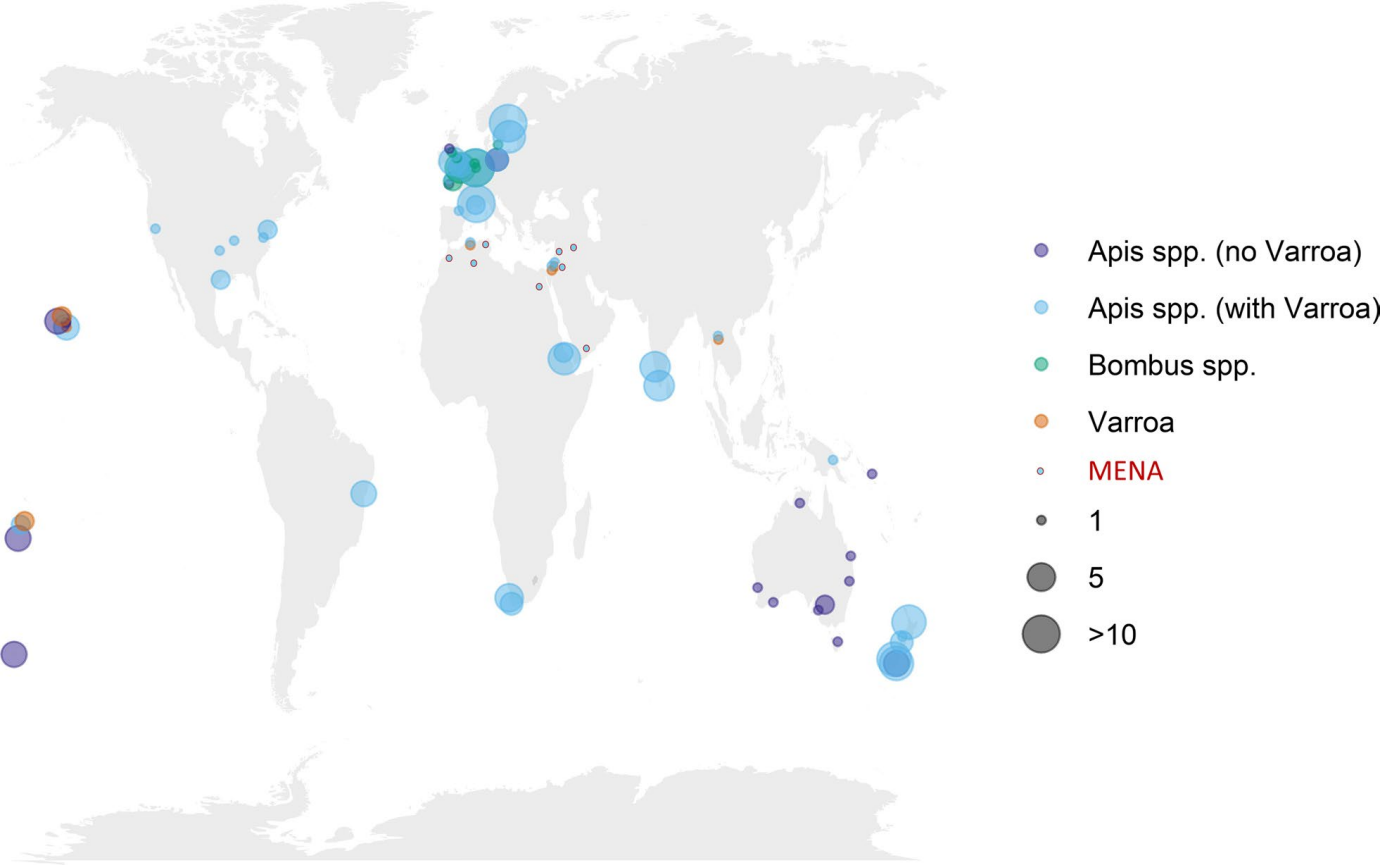
Host and geographic origin of screened samples (2 columns). Map showing the geographic and host origins of the samples and RNA sequencing libraries screened for the presence of DWV-D. Major host groups (*Apis, Bombus, Varroa*) and whether or not the samples come from areas free of varroa are represented by different colors. The number of unique SRAs of each type in each location is indicated by the size of the marker. The MENA samples screened by Sanger sequencing ([27]; Additional file 1: Fig. S2) are bounded by a red circle

To determine if DWV-D is currently still present as part of the larger assembly of DWV major and minor strains, we screened the raw, unprocessed reads of 300 published and unpublished RNA sequencing libraries from a wide geographic distribution of honeybee, varroa and bumblebee samples collected over the last 15 years (Fig. 2; Additional file 1: Table S3) for linked SNPs uniquely diagnostic for DWV-D. Since many reads will be common to all DWV strains, or subsets of strains, a bioinformatic strategy was designed to identify reads unique to just DWV-D, and distinct from DWV-A, -B and –C, to avoid potential misclassification of the read, and the sample. However, although many millions of DWV reads identified in these libraries, none was uniquely specific to DWV-D (Additional file 1: Table S3). We also looked more closely at the largest collection of DWV sequences from the Middle East and North Africa (MENA) region (68 samples), covering amino acids 10–73 of the Lp gene [27]. However, although a great diversity of minor amino acid polymorphs could be detected across the MENA region in this fragment, none were specific to DWV-D. Most minor polymorphs in the MENA samples were unique. Two were shared with DWV-B, -C and –D; two were shared with DWV-C and DWV-D; four were shared only with DWV-B, and two only with DWV-C (Additional file 1: Fig. 2). Moreover, while many DWV-B polymorphic variants were identified in the MENA collection, only a small fraction of the variants specific to DWV-C or DWV-C/DWV-D were identified. This implies that by 2012, when these samples were collected, the MENA region DWV profile was dominated by unique polymorphic amino acid variants clustered around DWV-A, some of which were also found in DWV-B, and no convincing evidence for the presence of either DWV-C or DWV-D.

## Discussion

Here, we have characterized the complete nucleotide sequence of a novel, fourth master variant of deformed wing virus, isolated from a sample of diseased honeybees collected in 1977 in Egypt. The virus is more closely related to DWV-C than to either DWV-A or DWV-B, clearly nested within the DWV group of variants across its entire genome, and with predicted genomic features implying that it should be functionally cross-process the other DWV variants polyproteins when coexisting within a common quasi-species.

Having identified this new major DWV-D variant in historical samples from Egypt collected around the time that *V. destructor* started to spread through the MENA region [68], the next logical question was to determine if DWV-D was still present, either in the MENA region or elsewhere. The strategy we chose was to screen a large selection of the thousands of honeybee, varroa and wild pollinator RNA sequencing libraries produced since 2005 for DWV-D. No assembled sequence similar to DWV-D has ever been reported from any of these RNA sequencing libraries. This suggests that DWV-D is currently very rare, or restricted either by region, host or historically. However, since the conventional bioinformatic pipelines only return assembled consensus sequences, which are then screened against the databases for identification, only the most common DWV variant present in any sample would have been identified. Currently, these are the DWV-A and DWV-B variants, or their recombinants, that are best adapted to transmission by *V. destructor* [21, 22, 45, 53, 71, 74] which is the primary driving force for the amount and genetic composition of DWV in honeybees and varroa [42, 60, 62, 68, 72, 74] and indirectly also in other pollinators in the vicinity of honeybee colonies, through secondary transmission [6, 19, 25, 37, 39, 72]. It is therefore possible that DWV-D is still part of the DWV quasi-species in these samples, but at too low a frequency to be identified by the conventional bioinformatic pipelines. It is also possible that DWV-D may be more readily identified in samples from the MENA region, or from geographic regions free from *V. destructor*, or in samples from non-*Apis* pollinators with possibly less dominance of the DWV-A and DWV-B variants associated with varroa-mediated transmission. We therefore preferentially selected libraries from the MENA region, from long-standing isolated honeybee populations worldwide (with and without varroa) and from non-Apis pollinators. Furthermore, the screening strategy focused on identifying SNPs uniquely specific for DWV-D, and distinct from DWV-A, -B and –C, in the individual sequence reads from these libraries, rather than assembled contigs. Since many of the reads are rather short (~ 50 nt) and furthermore contain random sequencing errors [24], the burden of proof for identifying DWV-D fell mostly on the Lp region, since this is the only region with a high enough SNP density to be able to identify on individual reads, several linked SNPs uniquely specific to DWV-D that could not be ascribed to either sequencing error or natural quasi-species variability. A similar strategy has been used previously to assign individual reads from publicly available RNA sequencing libraries to either DWV-A, DWV-B or DWV-C, with only conclusive evidence for the presence of DWV-A and DWV-B [10]. A qualitative analysis of minor amino acid polymorphisms for a large collection of samples from throughout the MENA region revealed that by 2012 the DWV profile in the MENA region was dominated by amino acid variants clustered around DWV-A, some of which were also found in DWV-B, with no convincing evidence for the presence of either DWV-C or DWV-D.

The fact that we could not find DWV-D in a range of samples from around the world does not necessarily mean that DWV-D has become extinct. It is impossible to conclusively prove the absence of something through negative screening, while only a single positive identification is required to prove DWV-D’s continued existence. However, it is not impossible for a virus, or a virus variant, to become extinct. Viruses are entirely dependent for their survival on the viability of their host populations. If these disappear before the virus has transmitted itself to other host populations, then the virus dies with these hosts and becomes effectively extinct. This process can be much more common than currently realized, especially if the virus (variant) developed in isolation and has a limited host range. Virus strains can also disappear more gradually from a quasi-species, through competition with better-adapted strains [40, 42, 61]. Although DWV can be detected in a wide range of insects, this is usually a secondary transmission in relation to a current, or historical, presence of infected Apis colonies in the area. The structural instability of the DWV particle in isolation [15, 65] precludes extensive viability and persistence outside the host tissues, such as in stored foods or the external environment. It also means that all the major DWV transmission routes involve immediate, close, and direct transfer of inoculum between tissues or through secretions [14, 72], which consequently requires a strong continuous presence of living hosts in close permanent contact with each other. In other words, the particularities of DWV make it a stronger candidate than more stable (ifla)viruses for the possible extinction of unique, locally evolved variants that are restricted by geography or host from wider dissemination and whose main host is *A. mellifera*, a managed bee species that itself is largely dependent on human activity for survival. That DWV-D may indeed have gone extinct is supported by the number of independent RNA libraries and the amount of effort expended searching for this variant, in current and historical samples (Fig. 2; Additional file 1: Table S3). The absence of DWV-D in the samples from around the world suggests this variant may indeed have been geographically restricted, and that if it is not extinct, then it would most likely be found in the geographic area where the original sample was obtained, either in local honeybees or the local wild pollinators or insects associated with beekeeping.

## Supplementary Information

**Additional file 1: Figure S1**. Amplification and sequencing strategy.** Figure S2**. MENA polymorphisms.** Table S1**. Primers for amplifying DWV strains.** Table S2**. Numerical details of the phylogenetic analyses.** Table S3**. SRA libraries and samples screened for DWV-D.

## Data Availability

The data generated and analysed during the current study are available in the GenBank repository under accession number MT504363. Original material is available from the corresponding author upon reasonable request.

## Abbreviations

3Cpro: 3C-protease
ABPV: Acute bee paralysis virus
bp: Basepair
BQCV: Black queen cell virus
CBPV: Chronic bee paralysis virus
cDNA: Complementary deoxyribonucleic acid
DNA: Deoxyribonucleic acid
DWV: Deformed wing virus
EBV: Egypt bee virus (re-assigned DWV strain-D)
Lp: L protein
KBV: Kashmir bee virus
MENA: Middle East & North Africa
nt: Nucleotide
UTR: Untranslated Region (5’ UTR; 3’ UTR)
ORF: Open reading frame
PCR: Polymerase chain reaction
RdRp: RNA dependent RNA polymerase
RNA: Ribonucleic acid
RT: Reverse transcription
SBPV: Slow bee paralysis virus
SBV: Sacbrood virus
SDS-PAGE: Sodium dodecyl sulphate poly acrylamide gel electrophoresis
SNP: Single nucleotide polymorphism
VDV-1: Varroa destructor virus-1 (re-assigned DWV strain-B)
VP: Viral protein (capsid protein)
Vpg: Viral protein genome-linked
