# Supplementary material for: Cold case: The disappearance of Egypt bee virus, a fourth distinct master strain of deformed wing virus linked to honeybee mortality in 1970’s Egypt
Source: Virol J. 2022 Jan 15;19:12. doi: 10.1186/s12985-022-01740-2 (PMC8760790; doi:10.1186/s12985-022-01740-2)
Supplement: Supplementary file 1 — Additional file 1: Figure S1. Amplification and sequencing strategy. Figure S2. MENA polymorphisms. Table S1. Primers for amplifying DWV strains. Table S2. Numerical details of the phylogenetic analyses. Table S3. SRA libraries and samples screened for DWV-D. [file 12985_2022_1740_MOESM1_ESM.pdf]

Supplementary files

Cold case: The disappearance of Egypt bee virus, a fourth distinct master strain of deformed wing virus linked to honeybee mortality in 1970's Egypt

Joachim R. de Miranda, Laura E. Brettell, Nor Chejanovsky, Anna K. Childers, Anne Dalmon, Ward Deboutte, Dirk C de Graaf, Vincent Doublet, Haftom Gebremedhn, Elke Genersch, Sebastian Gisder, Fredrik Granberg, Nizar J. Haddad, Rene Kaden, Robyn Manley, Jelle Matthijnssens, Ivan Meeus, Hussein Migdadi, Meghan O. Milbrath, Fanny Mondet, Emily J. Remnant, John M. K. Roberts, Eugene V. Ryabov, Noa Sela, Guy Smagghe, Hema Somanathan, Lena Wilfert, Owen N. Wright, Stephen J. Martin, Brenda V. Ball

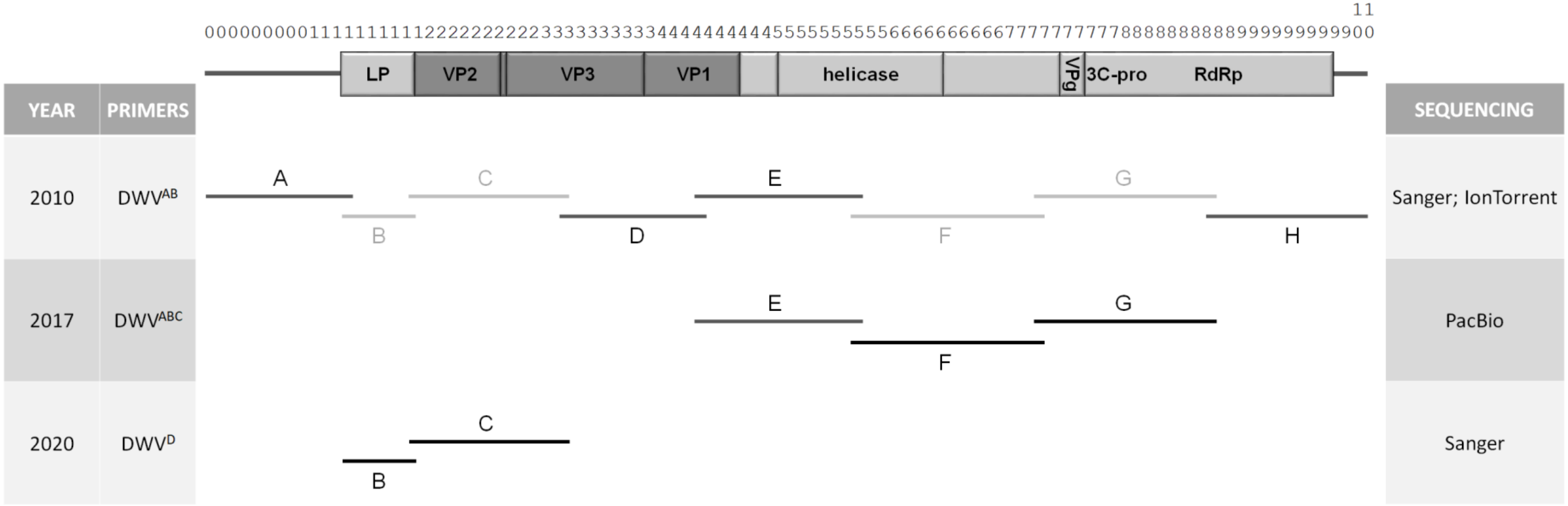

Supplementary Figure S1: Amplification and sequencing strategy

Amplification and sequencing strategy for determining the full DWV-D/EBV genome sequence. A genome map of DWV-D, indicating all the major structural and non-structural proteins, is shown together with a ruler in 100 nt intervals. Mapped onto this genome are the eight RT-PCR fragments (A-H) amplified with different sets of primers consensual for DWV-A, DWV-B and/or DWV-C, or specific for DWV-D. Also shown is the year the amplicons were produced and sequenced, as well as the primary sequencing technology used. Fragments in black were amplified successfully on the various occasions, while those in grey could not be amplified with the corresponding primers (Supplementary Table S1).

Amino Acid	10		12		14		16		18		20		22		24	
DWV-A	Y	S	A	V	A	Q	A	P	S	V	A	Y	A	P	R	T
VARIANT 2		A		A								H	T			S
VARIANT 3					P							C				
VARIANT 4												L				
VARIANT 5												R				
DWV-B		A										H				S
DWV-C		A			C							R		S	H	S
DWV-D		A			C							F		S	Q	S

Amino Acid	26		28		30		32		34		36		38		40	
DWV-A	W	E	V	D	E	A	R	R	R	R	V	I	K	R	L	A
VARIANT 2	C		I			G										
VARIANT 3			A			V										
VARIANT 4			S			P										
VARIANT 5																
DWV-B			I													
DWV-C			L	E		L			K							
DWV-D			L	E												

Amino Acid	42		44		46		48		50		52		54		56	
DWV-A	L	E	Q	E	R	I	R	N	V	L	D	V	G	V	Y	D
VARIANT 2	V					M	C	T				A	T			N
VARIANT 3										I		I	D			A
VARIANT 4													A			G
VARIANT 5																
DWV-B													T			
DWV-C						M			I	M		K	E		F	N
DWV-D						V			I	M			E		F	N

Amino Acid	58		60		62		64		66		68		70		72		Amino Acid
DWV-A	Q	A	T	W	E	Q	E	D	A	R	D	N	E	F	L	T	DWV-A
VARIANT 2		M					D	V	V				G			M	VARIANT 2
VARIANT 3																	VARIANT 3
VARIANT 4																	VARIANT 4
VARIANT 5																	VARIANT 5
DWV-B	H	T														M	DWV-B
DWV-C	D	L						G	V	E			V			L	DWV-C
DWV-D	N	L							V	K							DWV-D

Supplementary Figure S2: MENA polymorphisms

Minor variants for amino acids 10-73 of the Leader protein (Lp) identified among 68 samples from throughout the MENA region collected in 2012 (Haddad et al. 2017) as compared to the amino acid variants for DWV-B, DWV-C and DWV-D. The various colours and shades identify minor variants in the MENA region that were also found in DWV-B, DWV-C and DWV-D (light grey); in DWV-C and DWV-D (light purple); only in DWV-B (dark grey); only in DWV-C (light blue). Minor variants that are unique to just DWV-D are shown in light red.

Fragment	Primers (AB)	Sequence (5' to 3')	Size	Primers (ABC)	Sequence (5' to 3')	Size	Primers (D)	Sequence (5' to 3')	Size
A	DWV$^{(AB)}$-F27	CATAGCGAATTACGGTGC	1230	DWV$^{(ABC)}$-F27	CATAGCGAAGTACGGTGC	1226			
	DWV$^{(AB)}$-B1225	GCCGCCTAGCTTCATC		DWV$^{(ABC)}$-B1215	CCGTAATTCTTCAAGTTCCCA				
B	DWV$^{(AB)}$-F1153	ATTAAAAATGGCCTTTAGTTG	694	DWV$^{(ABC)}$-F1113	TTTTCAATTTAGTTTTGAATTTGAAGG	823	DWV$^{(D)}$-F1104	TTTTCAATTTAGTTTTGAATTTGAAGG	1334
	DWV$^{(AB)}$-B1806	CTTTTCTAATTCAACTTCACC		DWV$^{(ABC)}$-B1887	GTGGTCAAAACTACATTACTATC		DWV$^{(D)}$-B2403	AGCACTCATCCGCAAGGG	
C	DWV$^{(AB)}$-F1725	GATTACGAGTTAGAGTGTG	1407	DWV$^{(ABC)}$-F1719	AGCAGCGAGAATATGAGTTAG	1470	DWV$^{(D)}$-F2271	AAATGGATCATGCTTTAATTAGTG	860
	DWV$^{(AB)}$-B3095	GCTATTACTTTCCTGTAAATC		DWV$^{(ABC)}$-B3151	CATACTTACGCACCCACC		DWV$^{(D)}$-B3084	AAAACTATTGCTCTCTTGTAAATC	
D	DWV$^{(AB)}$-F3018	GTAGGTTAATTGTAGGTTATG	1344	DWV$^{(ABC)}$-F3018	AGGTAGGTTAATTGTAGGTTATG	1360			
	DWV$^{(AB)}$-B4329	CAATTCTATAACATTATTTACAC[illegible]		DWV$^{(ABC)}$-B4329	TTCTAATTCTATAACATTATTTACACG				
E	DWV$^{(AB)}$-F4220	TTTGGGTACAACATCGACC	1485	DWV$^{(ABC)}$-F4226	TACAACATCGTCCGGATCG	1536			
	DWV$^{(AB)}$-B5668	CACACTGATCCCAATAATC		DWV$^{(ABC)}$-B5722	TTGTAGATGTTTCAACACTCCA				
F	DWV$^{(AB)}$-F5625	ACGTGCGAGTCGTACTC	1706	DWV$^{(ABC)}$-F5670	ACCCGTTATCTGATTATTGGG	1688	DWV$^{(D)}$)-F5130	GATTGGGTAAAATTAGCTACAG	2464
	DWV$^{(AB)}$-B7295	ACGCAGTTATACCAATTATAC		DWV$^{(ABC)}$-B7295	GTACGCAGTTATACCAATTATAC		DWV$^{(D)}$-B7554	CTTTAGGAAACACCACGCC	
G	DWV$^{(AB)}$-F7243	CCCGTGAAAATGATTCGTG	1588	DWV$^{(ABC)}$-F7243	CCCGTGAAAATGATTCGTG	1610	DWV$^{(D)}$-F7834	GATAGTAACATTGTATTGGTGAC	965
	DWV$^{(AB)}$-B8794	CCGTGAATATAGTGTGAGG		DWV$^{(ABC)}$-B8811	CAAGCATGTATCTTTCAAACAATC		DWV$^{(D)}$-B8765	CTCATTAACTGCGTAGTTGAC	
H	DWV$^{(AB)}$-F8688	GGTAAGCGATGGTTGTTTG	1451	DWV$^{(ABC)}$-F8718	TGCAAGATTCGGGATGTTATC	1430			
	DWV$^{(AB)}$-B10101	ACTATACTAAAATTAGGACGC		DWV$^{(ABC)}$-B10102	GTTAAAACTATACTAAAATTAGGACG				

Supplementary Table S1: Primers for amplifying DWV strains

Primers used for amplifying and sequencing the DWV-D/EBV genome. Shown are the genome fragment (A-H), the primer names, sequences and product sizes for the three sets of primers developed: those consensual for DWV-A and DWB-B (left); those consensual for DWV-A, DWV-B and DWV-C (middle) and those specific for DWV-D/EBV (right).

MEGA-X		Genomic Region			
		UTR	Lp	VP	Non-Structural
ML estimate	nt	-4852.45	-2855.00	-11287.30	-19530.24
Characters	nt	1217	579	2789	4918
Taxa	nt	5	5	5	5
ML estimate	AA	*n.a*	-1506.96	-5007.60	-9614.84
Characters	AA	*n.a*	180	927	1659
Taxa	AA	*n.a*	5	5	5

Supplementary Table S2: Numerical details of the phylogenetic analyses

Numerical details of the phylogenetic analyses. Shown are the name of the genomic region studied; the Maximum Likelihood estimate for the most likely trees (shown in Figure 1); the number of characters included in the phylogenetic analyses and the number of taxa in the analyses. Abbreviations are: the 5’ and 3’ Untranslated Regions (UTR); the Lp protein (Lp); capsid proteins (VP) and the Non-Structural proteins. Analyses were conducted separately on the nucleotide (nt) and the amino acid (AA) sequences.

Sample-ID	Host	Subspecies	Year	Location	Country	Lat	Lon	Varroa	SRA	Reference	NGS	Length	Total Reads	Total DWV	DWV-D
62-V_A_h2	*Apis mellifera*	*n.d.*	2015	Big Island, Hawaii	USA	19,91	-155,99	Yes	SRX9383000	Brettell et al. 2020	HiSeq	150	13158865	2864	0
126-V_W_h3	*Apis mellifera*	*n.d.*	2016	Oahu, Hawaii	USA	19,26	-155,48	Yes	SRX9383069	Brettell et al. 2020	HiSeq	150	17565046	8662931	0
2-V_W_h1	*Apis mellifera*	*n.d.*	2015	Big Island, Hawaii	USA	21,32	-157,79	Yes	SRX9383040	Brettell et al. 2020	HiSeq	150	5057364	2368249	0
129-N_A_h6	*Apis mellifera*	*n.d.*	2016	Kauai, Hawaii	USA	19,49	-155,91	No	SRX9383072	Brettell et al. 2020	HiSeq	150	14167416	14267	0
43-N_A_h3	*Apis mellifera*	*n.d.*	2016	Kauai, Hawaii	USA	21,97	-159,40	No	SRX9383106	Brettell et al. 2020	HiSeq	150	9142048	1145	0
64-N_A_h4	*Apis mellifera*	*n.d.*	2016	Kauai, Hawaii	USA	21,09	-157,01	No	SRX9383002	Brettell et al. 2020	HiSeq	150	11654794	3866	0
65-N_W_h2	*Apis mellifera*	*n.d.*	2016	Kauai, Hawaii	USA	21,09	-157,01	No	SRX9383003	Brettell et al. 2020	HiSeq	150	12764894	2501	0
132-Var1	*Varroa destructor*	*n.d.*	2015	Oahu, Hawaii	USA	22,07	-159,66	Yes		unpublished	HiSeq	150	14592630	11216540	0
HB_S16	*Apis mellifera*	*n.d.*	2012	Big Island, Hawaii	USA	19,26	-155,48	Yes	SRX5654483	Brettell et al. 2019	HiSeq	100	12254583	34793	0
HB_S40	*Apis mellifera*	*n.d.*	2012	Big Island, Hawaii	USA	20,05	-155,67	Yes	ERS636116	Mordecai et al. 2016	HiSeq	100	14207573	11623322	0
V_S48	*Varroa destructor*	*n.d.*	2012	Oahu, Hawaii	USA	21,30	-157,82	Yes	ERS636115	Mordecai et al. 2016	HiSeq	100	35259211	31477364	0
V_S32	*Varroa destructor*	*n.d.*	2012	Big Island, Hawaii	USA	20,75	-156,00	Yes	SRX5654493	Brettell et al. 2019	HiSeq	100	13578530	12379171	0
HB_S1	*Apis mellifera*	*n.d.*	2012	Maui, Hawaii	USA	20,81	-156,29	No		unpublished	HiSeq	150	92175914	13	0
159-UK_T_1	*Apis mellifera*	*n.d.*	2016	England	UK	50,92	-1,75	Yes		unpublished	HiSeq	150	14195025	3940973	0
161-UK_T_2	*Apis mellifera*	*n.d.*	2016	Wales	UK	52,97	-4,06	Yes		unpublished	HiSeq	150	16000811	918635	0
160-UK_U_1	*Apis mellifera*	*n.d.*	2016	England	UK	51,48	-1,08	Yes		unpublished	HiSeq	150	19561395	5247778	0
163-UK_U_2	*Apis mellifera*	*n.d.*	2016	England	UK	51,02	-1,10	Yes		unpublished	HiSeq	150	15741639	8069083	0
145-UK_U_5	*Apis mellifera*	*n.d.*	2016	Wales	UK	51,61	-3,60	Yes		unpublished	HiSeq	150	23341207	6958930	0
146-UK_U_6	*Apis mellifera*	*n.d.*	2016	Wales	UK	53,16	-3,85	Yes		unpublished	HiSeq	150	30700527	24053242	0
157-USA_T_5	*Apis mellifera*	*n.d.*	2016	California	USA	39,21	-121,05	Yes		unpublished	HiSeq	150	6858756	3551	0
140-USA_T_6	*Apis mellifera*	*n.d.*	2016	Missouri	USA	36,88	-90,04	Yes		unpublished	HiSeq	150	9943641	621270	0
154-USA_U_8	*Apis mellifera*	*n.d.*	2016	Oklahoma	USA	34,92	-95,76	Yes		unpublished	HiSeq	150	9986613	4173	0
156-USA_U_9	*Apis mellifera*	*n.d.*	2016	Virginia	USA	37,49	-78,59	Yes		unpublished	HiSeq	150	10510097	3154161	0
134-AHB1	*Apis mellifera*	*Africanized*	2017	Cruz das Almas	Brazil	-12,66	-39,09	Yes		unpublished	HiSeq	150	23217356	20532	0
135-AHB2	*Apis mellifera*	*Africanized*	2017	Cruz das Almas	Brazil	-12,66	-39,09	Yes		unpublished	HiSeq	150	18205415	17578	0
136-AHB3	*Apis mellifera*	*Africanized*	2017	Cruz das Almas	Brazil	-12,66	-39,09	Yes		unpublished	HiSeq	150	12272355	48422	0
137-AHB4	*Apis mellifera*	*Africanized*	2017	Cruz das Almas	Brazil	-12,66	-39,09	Yes		unpublished	HiSeq	150	11169414	10507	0
AB3	*Apis mellifera*	*intermissa*	2013	Bleda	Algeria	36,51	2,78	Yes	PRJNA437730	Haddad et al. 2018	HiSeq	100	22438819	78055	0
AV6	*Varroa destructor*	-	2013	Bleda	Algeria	36,51	2,78	Yes	PRJNA437731	Haddad et al. 2018	HiSeq	100	35486417	14157	0
SB2	*Apis mellifera*	*syriaca*	2013	Maru	Jordan	32,61	35,90	Yes	PRJNA437728	Haddad et al. 2018	HiSeq	100	34851751	500509	0
SV5	*Varroa destructor*	-	2013	Maru	Jordan	32,61	35,90	Yes	PRJNA437729	Haddad et al. 2018	HiSeq	100	36692614	457153	0
IB1	*Apis mellifera*	*ligustica*	2016	Zrifin	Israel	31,96	34,84	Yes	PRJNA329428	Levin et al. 2019	HiSeq	100	31221496	594906	0
IV4	*Varroa destructor*	-	2016	Zrifin	Israel	31,96	34,84	Yes	PRJNA329427	Levin et al. 2019	HiSeq	100	30500539	759633	0
BCER	*Apis cerana*	*indica*	2016	Phrae	Thailand	18,20	100,10	Yes	PRJNA475853	Levin et al. 2019	HiSeq	100	84071155	0	0
VCER	*Varroa jacobsoni*	-	2016	Phrae	Thailand	18,20	100,10	Yes	PRJNA475855	Levin et al. 2019	HiSeq	100	127875113	0	0
Bp1_Ls	*Bombus pascuorum*	-	2019	East Flanders	Belgium	51,04	3,73	*n.a.*		unpublished	HiSeq PE	50	10082822	0	0
Bp2_Ls	*Bombus pascuorum*	-	2019	East Flanders	Belgium	51,06	3,73	*n.a.*		unpublished	HiSeq PE	50	11624213	0	0
Bp3_Ls	*Bombus pascuorum*	-	2019	East Flanders	Belgium	51,02	3,72	*n.a.*		unpublished	HiSeq PE	50	9110288	0	0
Bp4_Ls	*Bombus pascuorum*	-	2019	East Flanders	Belgium	51,03	3,73	*n.a.*		unpublished	HiSeq PE	50	10591507	0	0
Bp5_ls	*Bombus pascuorum*	-	2020	East Flanders	Belgium	50,94	3,13	*n.a.*		unpublished	HiSeq PE	50	9749758	0	0
Bp6_Ls	*Bombus pascuorum*	-	2019	East Flanders	Belgium	50,94	3,14	*n.a.*		unpublished	HiSeq PE	50	10657920	0	0

Bp7_Ls	*Bombus pascuorum*	-	2019	East Flanders	Belgium	50,83	3,69	*n.a.*		unpublished	HiSeq PE	50	11145742	0	0
Bp8_Ls	*Bombus pascuorum*	-	2019	East Flanders	Belgium	50,84	3,70	*n.a.*		unpublished	HiSeq PE	50	10282459	0	0
Bp17_Ls	*Bombus pascuorum*	-	2019	East Flanders	Belgium	51,14	3,59	*n.a.*		unpublished	HiSeq PE	50	8840780	0	0
Bp18_Ls	*Bombus pascuorum*	-	2019	East Flanders	Belgium	51,14	3,57	*n.a.*		unpublished	HiSeq PE	50	9723808	0	0
Bp19_Ls	*Bombus pascuorum*	-	2019	East Flanders	Belgium	50,93	3,59	*n.a.*		unpublished	HiSeq PE	50	9764973	0	0
Bp20_Ls	*Bombus pascuorum*	-	2019	East Flanders	Belgium	50,93	3,58	*n.a.*		unpublished	HiSeq PE	50	10332530	0	0
PBS3	*Bombus terrestris*	-	2015	Rearing facility	Belgium	51,13	4,89	*n.a.*		unpublished	HiSeq PE	50	1113410	17	0
PBS4	*Bombus terrestris*	-	2016	Rearing facility	Belgium	51,13	4,89	*n.a.*		unpublished	HiSeq PE	50	786193	0	0
PBS5	*Bombus terrestris*	-	2017	Rearing facility	Belgium	51,13	4,89	*n.a.*		unpublished	HiSeq PE	50	1123440	2	0
PBS6	*Bombus terrestris*	-	2018	Rearing facility	Belgium	51,13	4,89	*n.a.*		unpublished	HiSeq PE	50	1118470	63	0
DWVP0-2013	*Apis mellifera*	*carnica*	2013	Hohen Neuendorf	Germany	52,68	13,27	Yes	MH678671.1	Gisder et al. 2018	MiSeq V3	300	983318	81060	0
DWVP0-2016	*Apis mellifera*	*carnica*	2016	Hohen Neuendorf	Germany	52,68	13,27	Yes	MH678672.1	Gisder et al. 2018	MiSeq V3	300	581066	580178	0
DWVP0-2017	*Apis mellifera*	*carnica*	2017	Hohen Neuendorf	Germany	52,68	13,27	Yes	MH678673.1	Gisder et al. 2018	MiSeq V3	300	663602	656048	0
DWVP1-2014	*Apis mellifera*	*carnica*	2014	Hohen Neuendorf	Germany	52,68	13,27	No	MH678668.1	Gisder et al. 2018	MiSeq V3	300	1858182	173846	0
DWVP1-2016	*Apis mellifera*	*carnica*	2016	Hohen Neuendorf	Germany	52,68	13,27	No	MH678669.1	Gisder et al. 2018	MiSeq V3	300	639614	637288	0
DWVP1-2017	*Apis mellifera*	*carnica*	2017	Hohen Neuendorf	Germany	52,68	13,27	No	MH678670.1	Gisder et al. 2018	MiSeq V3	300	783942	773548	0
Am1	*Apis mellifera*	*n.d.*	2016	Berkshire	England	51,49	-1,17	Yes		unpublished	HiSeq	100	217719390	6623508	0
Am2	*Apis mellifera*	*n.d.*	2016	Berkshire	England	51,49	-1,17	Yes		unpublished	HiSeq	100	246032444	17335575	0
Am3	*Apis mellifera*	*n.d.*	2016	Berkshire	England	51,49	-1,17	Yes		unpublished	HiSeq	100	227050968	6841306	0
Bt1	*Bombus terrestris*	-	2016	Berkshire	England	51,49	-1,17	*n.a.*		unpublished	HiSeq	100	224331666	8	0
Bt2	*Bombus terrestris*	-	2016	Berkshire	England	51,49	-1,17	*n.a.*		unpublished	HiSeq	100	270180722	76	0
Bt3	*Bombus terrestris*	-	2016	Berkshire	England	51,49	-1,17	*n.a.*		unpublished	HiSeq	100	198937166	12	0
Bl1	*Bombus lapidarius*	-	2016	Berkshire	England	51,49	-1,17	*n.a.*		unpublished	HiSeq	100	254351158	71	0
Bl2	*Bombus lapidarius*	-	2016	Berkshire	England	51,49	-1,17	*n.a.*		unpublished	HiSeq	100	174248630	103	0
Bl3	*Bombus lapidarius*	-	2016	Berkshire	England	51,49	-1,17	*n.a.*		unpublished	HiSeq	100	232558976	90	0
Ma-S1	*Apis mellifera*	*n.d.*	2017	Provence	France	44,05	5,13	Yes		unpublished	DNBSEQ	100	12721044	11337251	0
Ma-S2	*Apis mellifera*	*n.d.*	2017	Provence	France	44,05	5,13	Yes		unpublished	DNBSEQ	100	14270082	9774020	0
Ma-S5	*Apis mellifera*	*n.d.*	2017	Provence	France	44,05	5,13	Yes		unpublished	DNBSEQ	100	16342718	4954143	0
Ma-S6	*Apis mellifera*	*n.d.*	2017	Provence	France	44,05	5,13	Yes		unpublished	DNBSEQ	100	18598430	36931	0
Bo-T1	*Apis mellifera*	*n.d.*	2017	Provence	France	43,84	4,88	Yes		unpublished	DNBSEQ	100	17611170	3537738	0
Bo-T3	*Apis mellifera*	*n.d.*	2017	Provence	France	43,84	4,88	Yes		unpublished	DNBSEQ	100	18285358	4014996	0
Ma-T1	*Apis mellifera*	*n.d.*	2017	Provence	France	44,05	5,13	Yes		unpublished	DNBSEQ	100	16638864	3907767	0
Ma-T5	*Apis mellifera*	*n.d.*	2017	Provence	France	44,05	5,13	Yes		unpublished	DNBSEQ	100	15211406	5363392	0
443	*Apis mellifera*	*n.d.*	2018	Provence	France	44,05	5,13	Yes		unpublished	DNBSEQ	100	15336914	7702253	0
404	*Apis mellifera*	*n.d.*	2018	Provence	France	44,05	5,13	Yes		unpublished	DNBSEQ	100	18218788	15128	0
418	*Apis mellifera*	*n.d.*	2018	Provence	France	44,05	5,13	Yes		unpublished	DNBSEQ	100	14865942	8401728	0
442	*Apis mellifera*	*n.d.*	2018	Provence	France	44,05	5,13	Yes		unpublished	DNBSEQ	100	15594840	6347730	0
NG-7006_C1	*Apis mellifera*	*n.d.*	2013	Warwick	England	52,38	-1,56	Yes	PRJEB5249	Ryabov et al. 2014	HiSeq	101	10069125	4269120	0
NG-7006_E7	*Apis mellifera*	*n.d.*	2013	Warwick	England	52,38	-1,56	Yes	PRJEB5249	Ryabov et al. 2014	HiSeq	101	8843179	6545702	0
B2_Hem	*Apis mellifera*	*n.d.*	2010	Texas, Maryland	USA	39,03	-76,92	Yes	SRP135682	Ryabov et al. 2017	HiSeq	101	25758539	13803868	0
B2_PBS	*Apis mellifera*	*n.d.*	2010	Texas	USA	29,23	-95,35	Yes	SRP135682	Ryabov et al. 2017	HiSeq	101	20353326	3906878	0
V31_PBS	*Apis mellifera*	*n.d.*	2016	Texas	USA	29,23	-95,35	Yes	SRP135682	Ryabov et al. 2017	HiSeq	150	14218987	71662	0
V99_VIROCT15	*Apis mellifera*	*n.d.*	2015	Maryland	USA	39,03	-76,92	Yes	SRP135682	Ryabov et al. 2017	HiSeq	150	14493557	14258748	0
SA_SB_C1	*Apis mellifera*	*capensis*	2013	Stellenbosch	South Africa	-33,95	18,84	Yes	SRX2422224	Remnant et al. 2017	HiSeq	100	89462246	17058	0
SA_SB_K2	*Apis mellifera*	*capensis*	2013	Stellenbosch	South Africa	-33,95	18,84	Yes	SRX2422218	Remnant et al. 2017	HiSeq	100	84044582	12677	0

SA_SB_A8	*Apis mellifera*	*capensis*	2013	Stellenbosch	South Africa	-33,95	18,84	Yes		unpublished	HiSeq	100	97631138	8768	0
SA_RI_A	*Apis mellifera*	*capensis*	2013	Robben Island	South Africa	-33,81	18,37	Yes	SRX2422212	Remnant et al. 2017	HiSeq	100	40214438	1571	0
SA_RI_11	*Apis mellifera*	*capensis*	2013	Robben Island	South Africa	-33,81	18,37	Yes	SRX2422225	Remnant et al. 2017	HiSeq	100	41030460	2358	0
SA_RI_49	*Apis mellifera*	*capensis*	2013	Robben Island	South Africa	-33,81	18,37	Yes	SRX2422223	Remnant et al. 2017	HiSeq	100	37640156	1642	0
SA_RI_48	*Apis mellifera*	*capensis*	2013	Robben Island	South Africa	-33,81	18,37	Yes		unpublished	HiSeq	100	34889486	556	0
SA_RI_IS7	*Apis mellifera*	*capensis*	2013	Robben Island	South Africa	-33,81	18,37	Yes		unpublished	HiSeq	100	32786158	62	0
T_E13	*Apis mellifera*	*n.d.*	2015	Eua	Tonga	-21,36	-174,97	No		unpublished	HiSeq	100	40443878	3390	0
T_E17	*Apis mellifera*	*n.d.*	2015	Eua	Tonga	-21,37	-174,95	No		unpublished	HiSeq	100	40610394	3352	0
T_T12	*Apis mellifera*	*n.d.*	2015	Tongatapu	Tonga	-21,15	-175,19	No	SRX2422220	Remnant et al. 2017	HiSeq	100	42283492	3135	0
T_T23	*Apis mellifera*	*n.d.*	2015	Tongatapu	Tonga	-21,13	-175,21	No	SRX2422226	Remnant et al. 2017	HiSeq	100	38406846	2253	0
T_V9	*Apis mellifera*	*n.d.*	2015	Vava'u	Tonga	-18,68	-173,99	Yes	SRX2422214	Remnant et al. 2017	HiSeq	100	37316706	10558772	0
T_V10	*Apis mellifera*	*n.d.*	2015	Vava'u	Tonga	-18,63	-173,95	Yes	SRX2422213	Remnant et al. 2017	HiSeq	100	39288984	7687224	0
T_varroa1	*Varroa destructor*	-	2015	Vava'u	Tonga	-18,61	-173,93	Yes		unpublished	HiSeq	100	30688748	25292413	0
T_varroa2	*Varroa destructor*	-	2015	Vava'u	Tonga	-18,65	-173,93	Yes		unpublished	HiSeq	100	31994442	23909818	0
NZ_G0	*Apis mellifera*	*n.d.*	2015	Hamilton	New Zealand	-37,77	175,31	Yes	SRX3069206	Remnant et al, 2019	HiSeq	100	39969370	35158900	0
NZ_154	*Apis mellifera*	*n.d.*	2013	Hamilton	New Zealand	-37,77	175,31	Yes		unpublished	HiSeq	100	78597230	20397701	0
NZ_267	*Apis mellifera*	*n.d.*	2013	Hamilton	New Zealand	-37,77	175,31	Yes		unpublished	HiSeq	100	84737240	54796927	0
NZ_315	*Apis mellifera*	*n.d.*	2013	Hamilton	New Zealand	-37,77	175,31	Yes		unpublished	HiSeq	100	90881398	43988834	0
2a	*Apis mellifera*	*n.d.*	2012	Dunedin	New Zealand	-45,86	170,33	No		unpublished	HiSeq	50	51664151	8	0
7b	*Apis mellifera*	*n.d.*	2012	Dunedin	New Zealand	-45,87	170,12	No		unpublished	HiSeq	50	51590120	1	0
12b	*Apis mellifera*	*n.d.*	2012	Dunedin	New Zealand	-45,97	170,25	No		unpublished	HiSeq	50	59039189	0	0
16a	*Apis mellifera*	*n.d.*	2012	Dunedin	New Zealand	-46,11	170,07	No		unpublished	HiSeq	50	65648275	2	0
21b	*Apis mellifera*	*n.d.*	2012	Chatham Islands	New Zealand	-44,10	-176,56	No		unpublished	HiSeq	50	62278207	0	0
27a	*Apis mellifera*	*n.d.*	2012	Chatham Islands	New Zealand	-44,02	-176,63	No		unpublished	HiSeq	50	54152981	0	0
28b	*Apis mellifera*	*n.d.*	2012	Chatham Islands	New Zealand	-43,97	-176,52	No		unpublished	HiSeq	50	51189322	0	0
35a	*Apis mellifera*	*n.d.*	2012	Chatham Islands	New Zealand	-44,01	-176,42	No		unpublished	HiSeq	50	59844057	0	0
100b	*Apis mellifera*	*n.d.*	2013	Dunedin	New Zealand	-45,86	170,33	Yes		unpublished	HiSeq	50	48487053	285	0
102b	*Apis mellifera*	*n.d.*	2013	Dunedin	New Zealand	-45,87	170,12	Yes		unpublished	HiSeq	50	57124362	105	0
104a	*Apis mellifera*	*n.d.*	2013	Dunedin	New Zealand	-45,97	170,25	Yes		unpublished	HiSeq	50	57084491	314	0
114b	*Apis mellifera*	*n.d.*	2013	Dunedin	New Zealand	-46,11	170,07	Yes		unpublished	HiSeq	50	53839359	0	0
61a	*Apis mellifera*	*n.d.*	2012	Central Otago	New Zealand	-45,01	169,35	Yes		unpublished	HiSeq	50	53974939	9524	0
70b	*Apis mellifera*	*n.d.*	2012	Central Otago	New Zealand	-44,98	169,48	Yes		unpublished	HiSeq	50	52533961	4304558	0
72b	*Apis mellifera*	*n.d.*	2012	Central Otago	New Zealand	-45,17	169,23	Yes		unpublished	HiSeq	50	48888288	3961642	0
73b	*Apis mellifera*	*n.d.*	2012	Central Otago	New Zealand	-45,10	169,36	Yes		unpublished	HiSeq	50	68788331	12564	0
117a	*Apis mellifera*	*n.d.*	2014	Dunedin	New Zealand	-45,86	170,33	Yes		unpublished	HiSeq	50	57562118	33847	0
118a	*Apis mellifera*	*n.d.*	2014	Dunedin	New Zealand	-45,87	170,12	Yes		unpublished	HiSeq	50	54640062	24912	0
120b	*Apis mellifera*	*n.d.*	2014	Dunedin	New Zealand	-45,97	170,25	Yes		unpublished	HiSeq	50	44972661	4308385	0
128b	*Apis mellifera*	*n.d.*	2014	Dunedin	New Zealand	-46,11	170,07	Yes		unpublished	HiSeq	50	64401775	15927035	0
139a	*Apis mellifera*	*n.d.*	2014	Central Otago	New Zealand	-45,01	169,35	Yes		unpublished	HiSeq	50	57010781	75906	0
140a	*Apis mellifera*	*n.d.*	2014	Central Otago	New Zealand	-44,98	169,48	Yes		unpublished	HiSeq	50	51351641	1431	0
152b	*Apis mellifera*	*n.d.*	2014	Central Otago	New Zealand	-45,17	169,23	Yes		unpublished	HiSeq	50	56534630	799492	0
154b	*Apis mellifera*	*n.d.*	2014	Central Otago	New Zealand	-45,10	169,36	Yes		unpublished	HiSeq	50	66488340	2851	0
43a	*Apis mellifera*	*n.d.*	2012	Nelson	New Zealand	-40,60	172,65	Yes		unpublished	HiSeq	50	61220680	1283	0
51a	*Apis mellifera*	*n.d.*	2012	Nelson	New Zealand	-41,18	172,69	Yes		unpublished	HiSeq	50	78280685	1255	0
56a	*Apis mellifera*	*n.d.*	2012	Nelson	New Zealand	-41,34	172,52	Yes		unpublished	HiSeq	50	34532762	1141	0
57a	*Apis mellifera*	*n.d.*	2012	Nelson	New Zealand	-41,65	172,33	Yes		unpublished	HiSeq	50	64551924	789336	0

77b	*Apis mellifera*	*n.d.*	2012	Hamilton	New Zealand	-37,24	175,58	Yes		unpublished	HiSeq	50	56741027	3253745	0
88b	*Apis mellifera*	*n.d.*	2012	Hamilton	New Zealand	-37,48	175,20	Yes		unpublished	HiSeq	50	60354065	134035	0
91b	*Apis mellifera*	*n.d.*	2012	Hamilton	New Zealand	-37,61	175,27	Yes		unpublished	HiSeq	50	52590736	5436	0
93b	*Apis mellifera*	*n.d.*	2012	Hamilton	New Zealand	-37,81	174, 90	Yes		unpublished	HiSeq	50	64970159	3772	0
NT	*Apis mellifera*	*n.d.*	2014	North Territory	Australia	-14,46	132,26	No	PRJNA357523	Roberts et al. 2017	HiSeq	100	13913678	991	0
QLD	*Apis mellifera*	*n.d.*	2014	Queensland	Australia	-24,84	152,31	No	PRJNA357523	Roberts et al. 2017	HiSeq	100	15221333	211	0
WA2	*Apis mellifera*	*n.d.*	2014	West Australia	Australia	-33,86	121,88	No	PRJNA357523	Roberts et al. 2017	HiSeq	100	23874121	141	0
WA1	*Apis mellifera*	*n.d.*	2013	West Australia	Australia	-30,98	115,70	No	PRJNA357523	Roberts et al. 2017	HiSeq	100	29197119	0	0
ROB13	*Apis mellifera*	*n.d.*	2013	SE Australia	Australia	-34,31	142,19	No	PRJNA357523	Roberts et al. 2017	HiSeq	100	28387691	116	0
ROB14	*Apis mellifera*	*n.d.*	2014	SE Australia	Australia	-34,31	142,19	No	PRJNA357523	Roberts et al. 2017	HiSeq	100	26056639	1	0
VN	*Apis mellifera*	*n.d.*	2014	SE Australia	Australia	-29,74	151,74	No	PRJNA357523	Roberts et al. 2017	HiSeq	101	23266592	4	0
RE14	*Apis mellifera*	*n.d.*	2014	South Australia	Australia	-35,38	139,57	No	PRJNA357523	Roberts et al. 2017	HiSeq	100	18247107	0	0
TAS	*Apis mellifera*	*n.d.*	2014	Tasmania	Australia	-41,58	147,18	No	PRJNA357523	Roberts et al. 2017	HiSeq	100	19492851	2	0
PAM	*Apis mellifera*	*n.d.*	2014	Highlands	PNG	-6,01	145,37	Yes	PRJNA357523	Roberts et al. 2020	HiSeq	100	24992379	69	0
SAM	*Apis mellifera*	*n.d.*	2014	Malaita	Solomon Isl.	-8,76	160,70	No	PRJNA357523	Roberts et al. 2020	HiSeq	100	29987684	665	0
CC-jul09	*Apis mellifera*	*n.d.*	2009	Gotland	Sweden	57,14	18,31	Yes	SRR7781384	Thaduri et al. 2018	Ion Torrent	200	39342326	54878	0
CC-aug09	*Apis mellifera*	*n.d.*	2009	Gotland	Sweden	57,14	18,31	Yes	SRR7781385	Thaduri et al. 2018	Ion Torrent	200	37427260	72654	0
CC-oct09	*Apis mellifera*	*n.d.*	2009	Gotland	Sweden	57,14	18,31	Yes	SRR7781383	Thaduri et al. 2018	Ion Torrent	200	43216586	426884	0
BC-jul09	*Apis mellifera*	*Bond*	2009	Gotland	Sweden	57,07	18,22	Yes	SRR7781388	Thaduri et al. 2018	Ion Torrent	200	42307887	844603	0
BC-aug09	*Apis mellifera*	*Bond*	2009	Gotland	Sweden	57,07	18,22	Yes	SRR7781389	Thaduri et al. 2018	Ion Torrent	200	37789206	205090	0
BC-oct09	*Apis mellifera*	*Bond*	2009	Gotland	Sweden	57,07	18,22	Yes	SRR7781386	Thaduri et al. 2018	Ion Torrent	200	43092165	348965	0
BC-may10	*Apis mellifera*	*Bond*	2010	Gotland	Sweden	57,07	18,22	Yes	SRR7781387	Thaduri et al. 2018	Ion Torrent	200	41297699	273009	0
MS-apr15	*Apis mellifera*	*n.d.*	2015	Uppsala	Sweden	59,82	17,66	Yes	SRX13254142	Thaduri et al. 2021	Ion S5XL	200	28527353	763	0
MS-jun15	*Apis mellifera*	*n.d.*	2015	Uppsala	Sweden	59,82	17,66	Yes	SRX13254143	Thaduri et al. 2021	Ion S5XL	200	30093353	40	0
MS-aug15	*Apis mellifera*	*n.d.*	2015	Uppsala	Sweden	59,82	17,66	Yes	SRX13254144	Thaduri et al. 2021	Ion S5XL	200	31120511	15429	0
MS-sep15	*Apis mellifera*	*n.d.*	2015	Uppsala	Sweden	59,82	17,66	Yes	SRX13254145	Thaduri et al. 2021	Ion S5XL	200	28612326	72009	0
MS-oct15	*Apis mellifera*	*n.d.*	2015	Uppsala	Sweden	59,82	17,66	Yes	SRX13254146	Thaduri et al. 2021	Ion S5XL	200	32478179	111455	0
MR-apr15	*Apis mellifera*	*Bond*	2015	Uppsala	Sweden	59,82	17,66	Yes	SRX13254138	Thaduri et al. 2021	Ion S5XL	200	30001744	12	0
MR-jun15	*Apis mellifera*	*Bond*	2015	Uppsala	Sweden	59,82	17,66	Yes	SRX13254139	Thaduri et al. 2021	Ion S5XL	200	27156406	54	0
MR-aug15	*Apis mellifera*	*Bond*	2015	Uppsala	Sweden	59,82	17,66	Yes	SRX13254140	Thaduri et al. 2021	Ion S5XL	200	30170963	348965	0
MR-sep15	*Apis mellifera*	*Bond*	2015	Uppsala	Sweden	59,82	17,66	Yes	SRX13254141	Thaduri et al. 2021	Ion S5XL	200	30365924	207120	0
MR-oct15	*Apis mellifera*	*Bond*	2015	Uppsala	Sweden	59,82	17,66	Yes	SRX13254142	Thaduri et al. 2021	Ion S5XL	200	31030845	320429	0
BB-BioBest	*Bombus terrestris*	-	2018	Rearing facility	Belgium	51,13	4,89	*n.a.*		unpublished	Ion S5XL	300	2615564	0	0
BB-Koppert	*Bombus terrestris*	-	2018	Rearing facility	Netherlands	51,98	4,46	*n.a.*		unpublished	Ion S5XL	300	2402352	0	0
BB-Sweden	*Bombus spp*	*8 species*	2018	Skåne	Sweden	55,61	13,63	*n.a.*		unpublished	Ion S5XL	300	2375547	1	0
4782 (RL2)	*Apis mellifera*	*n.d.*	2010	Navarra	Spain	42,70	-1,70	*n.a.*	SRX13269450/51	Granberg et al. 2013	454	~255	241960	0	0
AA24115	*Apis mellifera*	*n.d.*	2019	Peyad	India	8,52	77,02	Yes		unpublished	DNBseq	100	41838962	0	0
AA25373	*Apis cerana*	*indica*	2019	Peyad	India	8,52	77,02	Yes		unpublished	DNBseq	100	40807136	0	0
AA26343	*Apis mellifera*	*n.d.*	2019	Malayam	India	8,48	77,02	Yes		unpublished	DNBseq	100	41358769	2	0
AA25369	*Apis cerana*	*indica*	2019	Malayam	India	8,48	77,02	Yes		unpublished	DNBseq	100	34551307	1	0
AA24018	*Apis cerana*	*indica*	2019	Vithura	India	8,68	77,14	Yes		unpublished	DNBseq	100	37675280	0	0
AA25485	*Apis cerana*	*indica*	2019	Trivandrum	India	8,43	76,99	Yes		unpublished	DNBseq	100	48934633	0	0
AA23630	*Apis cerana*	*indica*	2019	Kadakkal	India	8,83	76,92	Yes		unpublished	DNBseq	100	37861683	0	0
AA26132	*Apis cerana*	*indica*	2019	Marthrandam	India	8,31	77,21	Yes		unpublished	DNBseq	100	37770079	3	0
AA23792	*Apis mellifera*	*n.d.*	2019	Alakode	India	12,19	75,46	Yes		unpublished	DNBseq	100	41221284	1	0
AA26422	*Apis cerana*	*indica*	2019	Alakode	India	12,19	75,46	Yes		unpublished	DNBseq	100	36368431	0	0

AA26141	*Apis mellifera*	*n.d.*	2019	Nallompuzha	India	12,30	75,37	Yes		unpublished	DNBseq	100	41359560	0	0
AA25882	*Apis cerana*	*indica*	2019	Nallompuzha	India	12,30	75,37	Yes		unpublished	DNBseq	100	32129942	0	0
BBt	*Bombus terrestris*	-	2015	Le Conquet	France	48,36	-4,77	*n.a.*		unpublished	MiSeq	250	431974	0	0
BHb	*Apis mellifera*	*n.d.*	2015	Le Conquet	France	48,36	-4,77	Yes		unpublished	MiSeq	250	288071	11528	0
LBt	*Bombus terrestris*	-	2015	Liverpool	UK	53,41	-2,99	*n.a.*		unpublished	MiSeq	250	585487	2	0
LHb	*Apis mellifera*	*n.d.*	2015	Liverpool	UK	53,41	-2,99	Yes		unpublished	MiSeq	250	320740	11277	0
MBt	*Bombus terrestris*	-	2015	Isle of Man	UK	54,15	-4,49	*n.a.*		unpublished	MiSeq	250	520299	0	0
MHb	*Apis mellifera*	*n.d.*	2015	Isle of Man	UK	54,15	-4,49	No		unpublished	MiSeq	250	422457	0	0
Ubt	*Bombus terrestris*	-	2015	Ouessant	France	48,46	-5,09	*n.a.*		unpublished	MiSeq	250	323970	0	0
UHb	*Apis mellifera*	*n.d.*	2015	Ouessant	France	48,46	-5,09	No		unpublished	MiSeq	250	258823	0	0
BP1	*Apis mellifera*	*n.d.*	2012	Flanders	Belgium	51,03	3,96	Yes	SRR10418397	Deboutte et al. 2020	NextSeq	150	5957498	4161	0
BP2	*Apis mellifera*	*n.d.*	2012	Flanders	Belgium	51,03	3,96	Yes	SRR10418396	Deboutte et al. 2020	NextSeq	150	23930704	6480	0
BP3	*Apis mellifera*	*n.d.*	2012	Flanders	Belgium	51,15	4,46	Yes	SRR10418383	Deboutte et al. 2020	NextSeq	150	6934898	208	0
BP4	*Apis mellifera*	*n.d.*	2012	Flanders	Belgium	51,15	4,46	Yes	SRR10418372	Deboutte et al. 2020	NextSeq	150	8468066	83164	0
BP5	*Apis mellifera*	*n.d.*	2012	Flanders	Belgium	50,90	4,10	Yes	SRR10418361	Deboutte et al. 2020	NextSeq	150	24903398	44385	0
BP6	*Apis mellifera*	*n.d.*	2012	Flanders	Belgium	51,18	3,54	Yes	SRR10418350	Deboutte et al. 2020	NextSeq	150	31654094	4298637	0
BP7	*Apis mellifera*	*n.d.*	2012	Flanders	Belgium	51,18	3,54	Yes	SRR10418339	Deboutte et al. 2020	NextSeq	150	34427402	1752343	0
BP8	*Apis mellifera*	*n.d.*	2012	Flanders	Belgium	50,90	4,10	Yes	SRR10418328	Deboutte et al. 2020	NextSeq	150	27288860	3342220	0
BP9	*Apis mellifera*	*n.d.*	2012	Flanders	Belgium	51,09	3,17	Yes	SRR10418317	Deboutte et al. 2020	NextSeq	150	7462686	2340907	0
BP10	*Apis mellifera*	*n.d.*	2012	Flanders	Belgium	51,09	3,17	Yes	SRR10418306	Deboutte et al. 2020	NextSeq	150	7882664	1205660	0
BP11	*Apis mellifera*	*n.d.*	2012	Flanders	Belgium	50,83	4,76	Yes	SRR10418395	Deboutte et al. 2020	NextSeq	150	7982402	49383	0
BP12	*Apis mellifera*	*n.d.*	2012	Flanders	Belgium	50,83	4,76	Yes	SRR10418392	Deboutte et al. 2020	NextSeq	150	7332184	974166	0
BP13	*Apis mellifera*	*n.d.*	2012	Flanders	Belgium	51,04	5,38	Yes	SRR10418391	Deboutte et al. 2020	NextSeq	150	25168634	9074	0
BP14	*Apis mellifera*	*n.d.*	2012	Flanders	Belgium	51,04	5,38	Yes	SRR10418390	Deboutte et al. 2020	NextSeq	150	31393490	3208315	0
BP15	*Apis mellifera*	*n.d.*	2012	Flanders	Belgium	50,87	5,30	Yes	SRR10418389	Deboutte et al. 2020	NextSeq	150	45010868	3047	0
BP16	*Apis mellifera*	*n.d.*	2012	Flanders	Belgium	50,87	5,30	Yes	SRR10418388	Deboutte et al. 2020	NextSeq	150	24146430	2040	0
BP17	*Apis mellifera*	*n.d.*	2012	Flanders	Belgium	51,02	4,85	Yes	SRR10418387	Deboutte et al. 2020	NextSeq	150	6343574	6234	0
BP18	*Apis mellifera*	*n.d.*	2012	Flanders	Belgium	51,02	4,85	Yes	SRR10418386	Deboutte et al. 2020	NextSeq	150	7604626	57442	0
BP19	*Apis mellifera*	*n.d.*	2012	Flanders	Belgium	51,13	3,89	Yes	SRR10418385	Deboutte et al. 2020	NextSeq	150	6504826	74207	0
BP20	*Apis mellifera*	*n.d.*	2012	Flanders	Belgium	51,13	3,89	Yes	SRR10418384	Deboutte et al. 2020	NextSeq	150	7244666	1399125	0
BP21	*Apis mellifera*	*n.d.*	2012	Flanders	Belgium	50,97	3,09	Yes	SRR10418382	Deboutte et al. 2020	NextSeq	150	13414762	1607436	0
BP22	*Apis mellifera*	*n.d.*	2012	Flanders	Belgium	51,11	3,16	Yes	SRR10418381	Deboutte et al. 2020	NextSeq	150	7419086	28297	0
BP23	*Apis mellifera*	*n.d.*	2012	Flanders	Belgium	51,21	4,76	Yes	SRR10418380	Deboutte et al. 2020	NextSeq	150	8536746	11146	0
BP24	*Apis mellifera*	*n.d.*	2012	Flanders	Belgium	51,21	4,76	Yes	SRR10418379	Deboutte et al. 2020	NextSeq	150	9292866	168166	0
BP25	*Apis mellifera*	*n.d.*	2012	Flanders	Belgium	50,89	3,46	Yes	SRR10418378	Deboutte et al. 2020	NextSeq	150	5853776	1379838	0
BP26	*Apis mellifera*	*n.d.*	2012	Flanders	Belgium	50,89	3,46	Yes	SRR10418377	Deboutte et al. 2020	NextSeq	150	5266818	1023184	0
BP27	*Apis mellifera*	*n.d.*	2012	Flanders	Belgium	51,03	5,65	Yes	SRR10418376	Deboutte et al. 2020	NextSeq	150	5462848	737278	0
BP28	*Apis mellifera*	*n.d.*	2012	Flanders	Belgium	51,03	5,65	Yes	SRR10418375	Deboutte et al. 2020	NextSeq	150	5680036	414005	0
BP29	*Apis mellifera*	*n.d.*	2012	Flanders	Belgium	50,94	4,74	Yes	SRR10418374	Deboutte et al. 2020	NextSeq	150	4909534	2679	0
BP30	*Apis mellifera*	*n.d.*	2012	Flanders	Belgium	50,97	4,33	Yes	SRR10418373	Deboutte et al. 2020	NextSeq	150	6501168	7000	0
BP31	*Apis mellifera*	*n.d.*	2012	Flanders	Belgium	51,20	5,03	Yes	SRR10418371	Deboutte et al. 2020	NextSeq	150	5704348	36474	0
BP32	*Apis mellifera*	*n.d.*	2012	Flanders	Belgium	51,18	3,09	Yes	SRR10418370	Deboutte et al. 2020	NextSeq	150	3939058	196001	0
BP33	*Apis mellifera*	*n.d.*	2012	Flanders	Belgium	51,18	3,09	Yes	SRR10418369	Deboutte et al. 2020	NextSeq	150	4827440	107	0
BP34	*Apis mellifera*	*n.d.*	2012	Flanders	Belgium	50,84	4,20	Yes	SRR10418368	Deboutte et al. 2020	NextSeq	150	5159966	1110538	0
BP35	*Apis mellifera*	*n.d.*	2012	Flanders	Belgium	51,02	5,28	Yes	SRR10418367	Deboutte et al. 2020	NextSeq	150	4052182	8	0
BP36	*Apis mellifera*	*n.d.*	2012	Flanders	Belgium	51,02	5,28	Yes	SRR10418366	Deboutte et al. 2020	NextSeq	150	6386304	762564	0

BP37	*Apis mellifera*	*n.d.*	2012	Flanders	Belgium	50,77	4,06	Yes	SRR10418365	Deboutte et al. 2020	NextSeq	150	6253256	1067713	0
BP38	*Apis mellifera*	*n.d.*	2012	Flanders	Belgium	50,88	3,72	Yes	SRR10418364	Deboutte et al. 2020	NextSeq	150	4937838	748084	0
BP39	*Apis mellifera*	*n.d.*	2012	Flanders	Belgium	51,23	5,31	Yes	SRR10418363	Deboutte et al. 2020	NextSeq	150	3207142	432130	0
BP40	*Apis mellifera*	*n.d.*	2012	Flanders	Belgium	51,01	3,89	Yes	SRR10418362	Deboutte et al. 2020	NextSeq	150	7279450	1076856	0
BP41	*Apis mellifera*	*n.d.*	2012	Flanders	Belgium	51,10	3,99	Yes	SRR10418360	Deboutte et al. 2020	NextSeq	150	6641140	1430252	0
BP42	*Apis mellifera*	*n.d.*	2012	Flanders	Belgium	51,03	4,10	Yes	SRR10418359	Deboutte et al. 2020	NextSeq	150	6443968	1465172	0
BP43	*Apis mellifera*	*n.d.*	2012	Flanders	Belgium	51,01	4,30	Yes	SRR10418358	Deboutte et al. 2020	NextSeq	150	3969986	484	0
BP44	*Apis mellifera*	*n.d.*	2012	Flanders	Belgium	51,07	3,87	Yes	SRR10418357	Deboutte et al. 2020	NextSeq	150	5337436	957360	0
BP45	*Apis mellifera*	*n.d.*	2012	Flanders	Belgium	51,18	4,13	Yes	SRR10418356	Deboutte et al. 2020	NextSeq	150	3420788	13336	0
BP46	*Apis mellifera*	*n.d.*	2012	Flanders	Belgium	50,90	5,10	Yes	SRR10418355	Deboutte et al. 2020	NextSeq	150	5715844	1669683	0
BP47	*Apis mellifera*	*n.d.*	2012	Flanders	Belgium	51,08	5,24	Yes	SRR10418354	Deboutte et al. 2020	NextSeq	150	4472114	869848	0
BP48	*Apis mellifera*	*n.d.*	2012	Flanders	Belgium	51,15	4,65	Yes	SRR10418353	Deboutte et al. 2020	NextSeq	150	5152982	25653	0
BP49	*Apis mellifera*	*n.d.*	2012	Flanders	Belgium	50,94	4,36	Yes	SRR10418352	Deboutte et al. 2020	NextSeq	150	6236962	1009708	0
BP50	*Apis mellifera*	*n.d.*	2012	Flanders	Belgium	51,09	3,90	Yes	SRR10418351	Deboutte et al. 2020	NextSeq	150	3668542	52	0
BP51	*Apis mellifera*	*n.d.*	2013	Flanders	Belgium	51,03	3,85	Yes	SRR10418349	Deboutte et al. 2020	NextSeq	150	9606954	1293464	0
BP52	*Apis mellifera*	*n.d.*	2013	Flanders	Belgium	51,03	3,85	Yes	SRR10418348	Deboutte et al. 2020	NextSeq	150	7817124	23065	0
BP53	*Apis mellifera*	*n.d.*	2013	Flanders	Belgium	51,09	4,24	Yes	SRR10418347	Deboutte et al. 2020	NextSeq	150	11204696	2134118	0
BP54	*Apis mellifera*	*n.d.*	2013	Flanders	Belgium	51,09	4,24	Yes	SRR10418346	Deboutte et al. 2020	NextSeq	150	10323126	1600214	0
BP55	*Apis mellifera*	*n.d.*	2013	Flanders	Belgium	51,16	4,67	Yes	SRR10418345	Deboutte et al. 2020	NextSeq	150	8567892	1135344	0
BP56	*Apis mellifera*	*n.d.*	2013	Flanders	Belgium	51,16	4,67	Yes	SRR10418344	Deboutte et al. 2020	NextSeq	150	9884910	164	0
BP57	*Apis mellifera*	*n.d.*	2013	Flanders	Belgium	50,84	2,72	Yes	SRR10418343	Deboutte et al. 2020	NextSeq	150	5580088	1977695	0
BP58	*Apis mellifera*	*n.d.*	2013	Flanders	Belgium	50,85	2,89	Yes	SRR10418342	Deboutte et al. 2020	NextSeq	150	11081704	19	0
BP59	*Apis mellifera*	*n.d.*	2013	Flanders	Belgium	51,16	4,67	Yes	SRR10418341	Deboutte et al. 2020	NextSeq	150	10011676	1530928	0
BP60	*Apis mellifera*	*n.d.*	2013	Flanders	Belgium	51,07	5,09	Yes	SRR10418340	Deboutte et al. 2020	NextSeq	150	10776852	321	0
BP61	*Apis mellifera*	*n.d.*	2013	Flanders	Belgium	51,09	4,24	Yes	SRR10418338	Deboutte et al. 2020	NextSeq	150	10587520	56896	0
BP62	*Apis mellifera*	*n.d.*	2013	Flanders	Belgium	51,33	4,60	Yes	SRR10418337	Deboutte et al. 2020	NextSeq	150	10204144	9370	0
BP63	*Apis mellifera*	*n.d.*	2013	Flanders	Belgium	50,97	4,98	Yes	SRR10418336	Deboutte et al. 2020	NextSeq	150	14462860	2396453	0
BP64	*Apis mellifera*	*n.d.*	2013	Flanders	Belgium	51,01	5,48	Yes	SRR10418335	Deboutte et al. 2020	NextSeq	150	11343930	71	0
BP65	*Apis mellifera*	*n.d.*	2013	Flanders	Belgium	51,01	5,48	Yes	SRR10418334	Deboutte et al. 2020	NextSeq	150	13681346	483901	0
BP66	*Apis mellifera*	*n.d.*	2013	Flanders	Belgium	50,94	4,57	Yes	SRR10418333	Deboutte et al. 2020	NextSeq	150	10806772	18276	0
BP67	*Apis mellifera*	*n.d.*	2013	Flanders	Belgium	50,94	4,57	Yes	SRR10418332	Deboutte et al. 2020	NextSeq	150	13994478	1823187	0
BP68	*Apis mellifera*	*n.d.*	2013	Flanders	Belgium	50,95	3,32	Yes	SRR10418331	Deboutte et al. 2020	NextSeq	150	12141518	1761289	0
BP69	*Apis mellifera*	*n.d.*	2013	Flanders	Belgium	51,22	4,89	Yes	SRR10418330	Deboutte et al. 2020	NextSeq	150	8022530	13894	0
BP70	*Apis mellifera*	*n.d.*	2013	Flanders	Belgium	51,22	4,89	Yes	SRR10418329	Deboutte et al. 2020	NextSeq	150	10609718	193	0
BP71	*Apis mellifera*	*n.d.*	2013	Flanders	Belgium	50,95	3,32	Yes	SRR10418327	Deboutte et al. 2020	NextSeq	150	10995850	1796000	0
BP72	*Apis mellifera*	*n.d.*	2013	Flanders	Belgium	51,00	3,88	Yes	SRR10418326	Deboutte et al. 2020	NextSeq	150	11861826	512550	0
BP73	*Apis mellifera*	*n.d.*	2013	Flanders	Belgium	51,00	3,88	Yes	SRR10418325	Deboutte et al. 2020	NextSeq	150	16180544	1041369	0
BP74	*Apis mellifera*	*n.d.*	2013	Flanders	Belgium	51,17	4,57	Yes	SRR10418324	Deboutte et al. 2020	NextSeq	150	10730000	5761811	0
BP75	*Apis mellifera*	*n.d.*	2013	Flanders	Belgium	51,22	4,40	Yes	SRR10418323	Deboutte et al. 2020	NextSeq	150	9564610	461	0
BP76	*Apis mellifera*	*n.d.*	2013	Flanders	Belgium	50,88	2,86	Yes	SRR10418322	Deboutte et al. 2020	NextSeq	150	10819630	788805	0
BP77	*Apis mellifera*	*n.d.*	2013	Flanders	Belgium	50,91	2,99	Yes	SRR10418321	Deboutte et al. 2020	NextSeq	150	9267018	1343548	0
BP78	*Apis mellifera*	*n.d.*	2013	Flanders	Belgium	51,04	5,52	Yes	SRR10418320	Deboutte et al. 2020	NextSeq	150	11254322	19	0
BP79	*Apis mellifera*	*n.d.*	2013	Flanders	Belgium	51,04	5,52	Yes	SRR10418319	Deboutte et al. 2020	NextSeq	150	11840988	468101	0
BP80	*Apis mellifera*	*n.d.*	2013	Flanders	Belgium	51,14	4,04	Yes	SRR10418318	Deboutte et al. 2020	NextSeq	150	9611514	5	0
BP81	*Apis mellifera*	*n.d.*	2013	Flanders	Belgium	51,14	4,04	Yes	SRR10418316	Deboutte et al. 2020	NextSeq	150	11399586	1730333	0
BP82	*Apis mellifera*	*n.d.*	2013	Flanders	Belgium	50,98	4,64	Yes	SRR10418315	Deboutte et al. 2020	NextSeq	150	13586622	2148691	0

BP83	*Apis mellifera*	*n.d.*	2013	Flanders	Belgium	50,97	4,75	Yes	SRR10418314	Deboutte et al. 2020	NextSeq	150	13162578	107004	0
BP84	*Apis mellifera*	*n.d.*	2013	Flanders	Belgium	51,07	3,02	Yes	SRR10418313	Deboutte et al. 2020	NextSeq	150	15432800	1812162	0
BP85	*Apis mellifera*	*n.d.*	2013	Flanders	Belgium	51,07	3,02	Yes	SRR10418312	Deboutte et al. 2020	NextSeq	150	8487678	271864	0
BP86	*Apis mellifera*	*n.d.*	2013	Flanders	Belgium	50,92	4,89	Yes	SRR10418311	Deboutte et al. 2020	NextSeq	150	12641522	1489160	0
BP87	*Apis mellifera*	*n.d.*	2013	Flanders	Belgium	50,92	4,95	Yes	SRR10418310	Deboutte et al. 2020	NextSeq	150	10219812	74394	0
BP88	*Apis mellifera*	*n.d.*	2013	Flanders	Belgium	51,00	4,86	Yes	SRR10418309	Deboutte et al. 2020	NextSeq	150	11120936	1489372	0
BP89	*Apis mellifera*	*n.d.*	2013	Flanders	Belgium	51,00	4,86	Yes	SRR10418308	Deboutte et al. 2020	NextSeq	150	10048238	1369780	0
BP90	*Apis mellifera*	*n.d.*	2013	Flanders	Belgium	51,11	4,82	Yes	SRR10418307	Deboutte et al. 2020	NextSeq	150	11915180	80961	0
BP91	*Apis mellifera*	*n.d.*	2013	Flanders	Belgium	51,11	4,82	Yes	SRR10418305	Deboutte et al. 2020	NextSeq	150	10934776	2548337	0
BP92	*Apis mellifera*	*n.d.*	2013	Flanders	Belgium	50,95	4,95	Yes	SRR10418304	Deboutte et al. 2020	NextSeq	150	14708578	664	0
BP93	*Apis mellifera*	*n.d.*	2013	Flanders	Belgium	50,85	3,83	Yes	SRR10418303	Deboutte et al. 2020	NextSeq	150	18333180	2663109	0
BP94	*Apis mellifera*	*n.d.*	2013	Flanders	Belgium	50,85	3,83	Yes	SRR10418302	Deboutte et al. 2020	NextSeq	150	20324886	15102228	0
BP95	*Apis mellifera*	*n.d.*	2013	Flanders	Belgium	50,85	3,27	Yes	SRR10418301	Deboutte et al. 2020	NextSeq	150	18832406	3246174	0
BP96	*Apis mellifera*	*n.d.*	2013	Flanders	Belgium	50,88	5,25	Yes	SRR10418300	Deboutte et al. 2020	NextSeq	150	20305402	2991931	0
BP97	*Apis mellifera*	*n.d.*	2013	Flanders	Belgium	50,91	5,03	Yes	SRR10418299	Deboutte et al. 2020	NextSeq	150	20892538	73721	0
BP98	*Apis mellifera*	*n.d.*	2013	Flanders	Belgium	51,00	3,46	Yes	SRR10418298	Deboutte et al. 2020	NextSeq	150	21245764	3192277	0
BP99	*Apis mellifera*	*n.d.*	2013	Flanders	Belgium	50,95	3,59	Yes	SRR10418297	Deboutte et al. 2020	NextSeq	150	10742598	15394	0
BP100	*Apis mellifera*	*n.d.*	2013	Flanders	Belgium	50,84	4,02	Yes	SRR10418296	Deboutte et al. 2020	NextSeq	150	17187642	25	0
BP101	*Apis mellifera*	*n.d.*	2013	Flanders	Belgium	50,83	5,20	Yes	SRR10418394	Deboutte et al. 2020	NextSeq	150	15290220	23358	0
BP102	*Apis mellifera*	*n.d.*	2013	Flanders	Belgium	50,83	5,20	Yes	SRR10418393	Deboutte et al. 2020	NextSeq	150	20733422	3737104	0
Ethi_1	*Apis mellifera*	*Simensis*	2017	Ganta Afeshum	Ethiopia	14,23	39,50	Yes	PRJNA559070	Gebremedhm et al. 2020	NextSeq	150	7581498	155074	0
Ethi_2	*Apis mellifera*	*Simensis*	2017	Kafta Humera	Ethiopia	13,99	36,85	Yes	PRJNA559070	Gebremedhm et al. 2020	NextSeq	150	6540078	138	0
Ethi_3	*Apis mellifera*	*Simensis*	2017	Degua Tembien	Ethiopia	13,67	39,24	Yes	PRJNA559070	Gebremedhm et al. 2020	NextSeq	150	7786998	58	0
Ethi_4	*Apis mellifera*	*Simensis*	2017	Kilete Awlealo	Ethiopia	13,80	39,61	Yes	PRJNA559070	Gebremedhm et al. 2020	NextSeq	150	8414120	0	0
Ethi_5	*Apis mellifera*	*Simensis*	2017	Kilete Awlealo	Ethiopia	13,75	39,55	Yes	PRJNA559070	Gebremedhm et al. 2020	NextSeq	150	7328122	6872	0
Ethi_6	*Apis mellifera*	*Simensis*	2017	Mekelle	Ethiopia	13,52	39,51	Yes	PRJNA559070	Gebremedhm et al. 2020	NextSeq	150	7754530	476270	0
Ethi_7	*Apis mellifera*	*Simensis*	2017	Hawzen	Ethiopia	13,99	39,42	Yes	PRJNA559070	Gebremedhm et al. 2020	NextSeq	150	6853040	327	0
Ethi_8	*Apis mellifera*	*Simensis*	2017	Atsy Wonberta	Ethiopia	13,75	39,71	Yes	PRJNA559070	Gebremedhm et al. 2020	NextSeq	150	7397012	905699	0
Ethi_9	*Apis mellifera*	*Simensis*	2017	Atsy Wonberta	Ethiopia	13,82	39,72	Yes	PRJNA559070	Gebremedhm et al. 2020	NextSeq	150	6907528	802649	0
Ethi_10	*Apis mellifera*	*Simensis*	2017	Gulo Mkada	Ethiopia	13,42	39,37	Yes	PRJNA559070	Gebremedhm et al. 2020	NextSeq	150	8578176	152	0

Supplementary Table S3: SRA libraries and samples screened for DWV-D

Details of the RNA sequencing libraries screened for the presence of DWV-D, including the year, geographic location and host organism the RNA was isolated from, the presence or not of *V. destructor* in the region, the sequencing technology used (NGS) and average read length, accession number (if available) of the Small Read Archive (SRA) where the original raw data is stored, the original reference (if available), the total number of raw reads screened, the number of these matching DWV in general, and the number matching uniquely to only DWV-D.
